# Physical exercise attenuates age‐related muscle atrophy and exhibits anti‐ageing effects via the adiponectin receptor 1 signalling

**DOI:** 10.1002/jcsm.13257

**Published:** 2023-05-24

**Authors:** Yuan‐Li Chen, Yi‐Cheng Ma, Jie Tang, Dan Zhang, Qiu Zhao, Jian‐Jun Liu, Hong‐Shu Tang, Jin‐Yu Zhang, Guang‐Hui He, Chi‐Hui Zhong, Yu‐Tong Wu, Heng‐Ruo Wen, Lan‐Qing Ma, Cheng‐Gang Zou

**Affiliations:** ^1^ State Key Laboratory for Conservation and Utilization of Bio‐Resources in Yunnan, School of Life Sciences Yunnan University Kunming Yunnan China; ^2^ Faculty of Basic Medicine Kunming Medical University Kunming Yunnan China; ^3^ Institute of Medical Biology Chinese Academy of Medical Sciences and Peking Union Medical College Kunming Yunnan China; ^4^ Department of Digestive Diseases, The First Affiliated Hospital Kunming Medical University Kunming Yunnan China; ^5^ Institute of Biomedical Engineering Kunming Medical University Kunming Yunnan China

**Keywords:** AdipoR1, autophagy, *C. elegans*, exercise, FOXO, rodent, skeletal muscle loss

## Abstract

**Background:**

Although the adiponectin signalling exerts exercise‐mimicking effects, whether this pathway contributes to the anti‐ageing benefits of physical exercise has not been established yet.

**Methods:**

Swim exercise training and wheel running were used to measure lifespan in the nematode *Caenorhabditis elegans* and skeletal muscle quality in mice, respectively. Muscle weight, muscle fibre cross‐sectional area (CSA) and myonuclei number were used to evaluate muscle mass. RNA sequencing (RNA‐Seq) analysis of skeletal muscle in exercised mice was used to study the underlying mechanisms. Western blot and immunofluorescence were performed to explore autophagy‐ and senescence‐related markers.

**Results:**

The *C. elegans* adiponectin receptor PAQR‐1/AdipoR1, but not PAQR‐2/AdipoR2, was activated (3.55‐fold and 3.48‐fold increases in p‐AMPK on Days 1 and 6, respectively, *P* < 0.001), which was involved in lifespan extension in exercised worms. Exercise training increased skeletal muscle mass index (1.29‐fold, *P* < 0.01), muscle weight (1.75‐fold, *P* < 0.001), myonuclei number (1.33‐fold, *P* < 0.05), muscle fibre CSA (1.39‐fold, *P* < 0.05) and capillary abundance (2.19‐fold, *P* < 0.001 for capillary density; 1.58‐fold, *P* < 0.01 for capillary number) in aged mice. Physical exercise reduced protein (2.94‐fold, *P* < 0.001) and mRNA levels (1.70‐fold, *P* < 0.001) of p16^INK4a^, a marker for cellular senescence, in skeletal muscle of aged mice. These beneficial effects of exercise on skeletal muscle of mice were dependent on AdipoR1. Kyoto Encyclopedia of Genes and Genomes (KEGG) analysis for differentially expressed genes in skeletal muscle between exercised mice with and without AdipoR1 knockdown by RNA‐Seq analysis revealed that several KEGG pathways, such as ‘AMPK signalling pathway’ (*P* < 0.001), ‘FOXO signalling pathway’ (*P* < 0.001) and ‘autophagy’ (*P* < 0.001) were overrepresented. Knockdown of *FoxO3a* inhibited exercise‐mediated beneficial effects on skeletal muscle quality of mice by inhibiting autophagy/mitophagy (3.81‐fold reduction in LC3‐II protein, *P* < 0.001; 1.53‐fold reduction in BNIP3 protein, *P* < 0.05). Knockdown of *daf‐16*, the FoxO homologue in *C. elegans* , reduced autophagy (2.77‐fold and 2.06‐fold reduction in GFP::LGG‐1 puncta in seam cells and the intestine, respectively, *P* < 0.05) and blocked lifespan extension by exercise in worms.

**Conclusions:**

Our findings provide insights into how the AdipoR1 pathway has an impact on the anti‐ageing benefits of exercise and implicate that activation of the AdipoR1 signalling may represent a potential therapeutic strategy for reducing age‐related loss of skeletal muscle.

## Introduction

Exercise is one of the most effective approaches to confer beneficial effects on healthspan and longevity.^1^ For instance, regular exercise can alleviate muscle atrophy, which is characterized by a progressive decline in muscle mass, quality and function with age.^2^ Currently, treadmill running and voluntary wheel running are the most common approaches to study physiological adaptation associated with exercise in rodent model.^3^ During the last decade, other model organisms, such as the fruit fly *Drosophila* and the nematode *Caenorhabditis elegans* , have become promising models for understanding whole‐organism effects of exercise on longevity.^4^, ^5^

Autophagy is a housekeeping mechanism that maintains intracellular homeostasis by degrading dysfunctional molecules and organelles.^6^ Increased autophagy in skeletal muscle is required for exercise‐induced metabolic adaptations, including promotion of muscle growth.^7^, ^8^ In mammals and worms, TFEB/HLH‐30,^9^ NRF2/SKN‐1^10^ and FOXO/DAF‐16^11^ are major transcription factors that regulate autophagy genes. Although exercise activates TFEB by promoting its nuclear translocation, neither overexpression nor deletion of TFEB influences autophagic activity in skeletal muscle of mice. Exercise upregulates the protein levels of FOXO3a, which is accompanied by activation of AMP‐activated protein kinase (AMPK) in skeletal muscle.^12^ Unlike FOXO1 or FOXO4, FOXO3a can be phosphorylated by AMPK on multiple sites.^13^ In addition, exercise induces mitophagy in skeletal muscle in an AMPK‐dependent manner.^14^ These findings implicate that FOXO3a is the potential downstream molecule of the AMPK signalling, probably contributing to exercise‐induced autophagy/mitophagy. To date, the molecular mechanism underlying exercise‐ induced autophagy/mitophagy is not completely elucidated.

In mammals, adiponectin exerts its beneficial effects on insulin sensitivity and energy expenditure via two homologous adiponectin receptors AdipoR1 and AdipoR2.^15^ AdipoR1 activates PGC‐1α by the AMPK/SIRT1 signalling, whereas AdipoR2 activates the PPARα pathway.^15^ In addition, the adiponectin signalling is involved in the regulation of lifespan.^16^ Our recent study has demonstrated that the activation of the PAQR‐2/AdipoR2 signalling by low temperature extends the lifespan of *C. elegans* by inducing autophagy.^16^ Intriguingly, the adiponectin pathways can mimic many of the metabolic effects of exercise.^17^ Notably, adiponectin plays a role in mediating the effects of exercise on hippocampal neurogenesis and depression via AdipoR1.^18^ However, whether the adiponectin pathways are involved in the anti‐ageing benefits of exercise has remained unexplored. Here, we took advantage of two established exercise paradigms to investigate the role of the adiponectin signalling in extending lifespan and alleviating muscle atrophy by exercise in worms and mice, respectively.

## Materials and methods

### Statement of ethics

The Animal Care and Use Committee of Kunming Medical University approved the procedures for mice studies, care and maintenance (Permit No. kmmu2021520). The investigation of our protocol conforms to the Guide for the Care and Use of Laboratory Animals, published by the US National Institutes of Health (NIH).

### Nematode strains and maintenance

N2 Bristol was used as the wild‐type strain. The mutant strains, including *daf‐16(mu86)*, *aak‐2(ok524)*, *paqr‐1(tm3262)*, *paqr‐2(tm3410)*, *stEx30[myo‐3p::GFP::myo‐3+rol‐6 (su1006)]*, *sqIs11[lgg‐1p::mCherry::GFP::lgg‐1+rol‐6]* and *zIs356[daf‐16p::daf‐16a/b::GFP+rol‐6(su1006)]*, were obtained from Caenorhabditis Genetics Center (CGC; http://www.cbs.umn.edu/CGC), funded by NIH Office of Research Infrastructure Programs (P40 OD010440). The strains *waEx15[fat‐7::GFP+lin15(+)]* and *hqEx476[hsp‐16.2p::nCherry; dod‐3p::gfp; mtl‐1p::bfp, unc‐119(+)]* were kindly provided by Drs. Bin Liang (Yunnan University) and Mengqiu Dong (National Institute of Biological Sciences, China), respectively. Worms were cultured on nematode growth medium (NGM) and fed on *Escherichi coli* OP50 at 20°C.^19^

### Mice

Male C57BL/6J mice (10–12 months of age) were obtained from the Nanjing Biomedical Research Institute (Licence No. SCXK [S] 2005‐0019). The mice were maintained under a 12‐h light/12‐h dark cycle with free access to food and water. The temperature and humidity in the room were maintained at 20 ± 2°C and 50 ± 5%, respectively. These mice were bred and housed until 16 months of age.^20^

### The wheel running exercise regimen

At the age of 16 months, all mice were acclimated to a motorized running‐wheel apparatus (#YLS‐10B, Yiyan, Jinan, China) (0.6 m/revolution, 15 rpm for 30 min every other day) for 3 days prior to beginning the training protocol. Each four animals were kept in a separate cage, whereas the running‐wheel apparatus was outside the cage. Before exercise started, each mouse was taken out of the cage and placed on a wheel to run. For long‐term exercise training, mice were trained at 20 rpm for 30 min/day, 5 days/week for 16 weeks. It should be noted that at a speed of 20 rpm, the total distance of each exercise session (30 min) is 320 m, and the intensity is 11 m/min. There are no published reference standards for intensity of forced wheel running. The standardization of treadmill exercise is classified by three different intensities as follows: low intensity (<15 m/min), moderate intensity (15–20 m/min) and high intensity (>20 m/min).^21^ Thus, in the current study, the intensity of 12 m/min represents low intensity. The animals were euthanized 1 day after the end of the last exercise session. The regions of the GAS muscle used for each assay were described in *Figure* *S1*.

### Swim exercise protocols

Swim exercise training was carried out as previously reported.^5^ Briefly, mid‐L4 worms were divided randomly into exercise and control groups. The worms in the exercise group were transformed to M9 buffer covering unseeded NGM plates, whereas the worms in the control group were transformed to unseeded NGM plates. After 90 min, the worms in both groups were returned to NGM plates seeded with *E. coli* OP50. The worms were given swim exercise training once a day for 8 days.

### RNA sequencing analysis

After 16 weeks of exercise, gastrocnemius muscles of mice were collected and frozen immediately in liquid nitrogen followed by storage at −80°C. Then the samples were sent to Seqhealth Technology Co. (Wuhan, China) for RNA sequencing (RNA‐Seq) analysis. *P* ≤ 0.05 and fold change ≥2 were used as a threshold for differential expression. For Gene Ontology enrichment analysis, we searched for enrichment of Kyoto Encyclopedia of Genes and Genomes (KEGG) pathway gene sets of the muscle transcriptomic datasets using DAVID. The Gene Expression Omnibus (GEO) accession number is GSE205019.

### RNA interference for worms

Bacterial RNAi clones were obtained from the Ahringer RNAi library. *E. coli* strain HT115 containing dsRNA‐expressing plasmids were cultured for 8 h with shaking in Luria Broth (LB) medium with 100‐μg/mL ampicillin at 37°C and then seeded onto NGM plates containing 100‐μg/mL ampicillin and 5‐mM isopropyl 1‐thio‐β‐d‐galactopyranoside (IPTG) to induce the expression of dsRNA for overnight at 25°C. L1 larvae were placed on these plates at 20°C until they reached L4 larvae stage.

### Western blotting

Worms or muscle tissues of mice were homogenized in liquid nitrogen and then resuspended in radioimmunoprecipitation assay (RIPA) buffer (#R0278, Sigma‐Aldrich, Shanghai, China) on ice for 1 h. Thirty micrograms of total protein lysates per sample was loaded and separated onto 10% to 12% sodium dodecyl sulfate–polyacrylamide gel electrophoresis (SDS‐PAGE) gel. Then proteins were transferred to polyvinylidene fluoride (PVDF) membranes (Millipore, Bedford, MA). The primary antibodies used were anti‐GFP (#M20004, Abmart Inc., Shanghai, China), anti‐LC3B (#83506, Cell Signaling, Shanghai, China), anti‐AMPK (#ab3759, Abcam, Cambridge, MA), anti‐β‐actin (#ab14128, Abcam), anti‐phospho‐AMPK (Thr172) (#2531, Cell Signaling), anti‐adiponectin (#ab22554, Abcam), anti‐AdipoR1 (#ab70362, Abcam), anti‐AdipoR1 (#ab77612, Abcam), anti‐APPL1 (#3858, Cell Signaling), anti‐APPL2 (14294‐1‐AP, Proteintech, Wuhan, China), anti‐phospho‐Ca^2+^/calmodulin‐dependent protein kinase kinase‐β (CaMKKβ) (Ser511) (#12818, Cell Signaling), anti‐CaMKKβ (11549‐1‐AP, Proteintech), anti‐phospho‐LKB1 (Ser428) (#3482, Cell Signaling), anti‐LKB1 (#3047, Cell Signaling) and anti‐p62/SQSTM1 (#sc‐48402, Santa Cruz Biotechnology, Santa Cruz, CA). The secondary antibodies used in our experiments were horseradish peroxidase (HRP)‐conjugated anti‐mouse (#A21010, Abbkine, Wuhan, China) or anti‐rabbit IgG (#A21020, Abbkine). The protein bands were detected using ECL (#32109, Thermo Fisher Scientific, Waltham, MA) on Amersham Imager 600 (GE HealthCare). Subsequent image analysis was performed using ImageJ software. Uncropped scans of all blots are provided in *Data S1*.

### Quantitative real‐time PCR

Total RNA was isolated using TRIzol reagent (#10296010, Invitrogen, Shanghai, China). cDNA was generated using a first‐strand cDNA synthesis kit (#KR118, Tiangen, Beijing, China). SYBR Green (#RR820A, Takara, Dalian, China) and Roche LightCycler 480 System (Roche Applied Science, Mannheim, Germany) were utilized for quantitative real‐time PCR (qPCR). 2^−ΔΔCT^ method was used to calculate relative expression normalized to an internal control of *C. elegans* *act‐1* or mouse β‐actin. The primers are listed in *Table* *S1*.

### Knockdown of *AdipoR1* and *FoxO3a* by short hairpin RNA in mice

Adeno‐associated virus serotype‐8 carrying short hairpin RNA (shRNA) against *AdipoR1* (sequence: 5′‐GGG ATT GCT CTA CTG ATT ATG‐3′), *FoxO3a* (sequence: 5′‐CAG CCG TGC CTT GTC AAA TTC‐3′) and a scrambled shRNA control (sequence: 5′‐CCT AAG GTT AAG TCG CCC TCG‐3′) were created and packaged by Ubigene Biosciences (Guangzhou, China). Mice were infected with 1 × 10^8^ PFU of adenovirus particles containing vectors expressing shRNA‐*AdipoR1*, shRNA‐*FoxO3a* or shRNA control through tail vein injection. The mice were allowed to recover in a warm cage and subjected to wheel running exercise at Day 3 after injection.

### Haematoxylin and eosin staining

Muscle tissues were collected and quickly frozen in liquid nitrogen for the determination of gene expression. The remaining part of the tissues was fixed in formalin, dehydrated in graded ethanol and embedded in paraffin. The transverse gastrocnemius muscle slices were stained with haematoxylin and eosin (HE). Images were captured using a Zeiss Axioskop 2 plus fluorescence microscope (Carl Zeiss, Jena, Germany). ImageJ was used to calculate muscle fibre cross‐sectional area (CSA) and myonuclei number.

### Immunohistochemical analysis

The paraformaldehyde‐fixed paraffin‐embedded muscle sections were blocked by 3% bovine serum albumin (BSA)–phosphate‐buffered saline (PBS) solution for 1 h. Then the sections were stained with anti‐BNIP3 antibody (#ab10433, Abcam) overnight at 4°C. After rinsed with PBS three times, the sections were incubated with HRP‐conjugated goat anti‐mouse IgG (#ab205719, Abcam) for 1 h at room temperature. Then the substrate solution diaminobenzidine (#PA110, Tiangen) was added into the sections for colour development. Then the sections were counterstained with haematoxylin. ImageJ software was used to determine the integrated optical density per stained area for the positive staining.

### Immunofluorescence staining

After blocking with 10% goat serum in PBS overnight, the paraformaldehyde‐fixed paraffin‐embedded muscle sections were incubated with anti‐CD31 (#SAB5700639, Sigma‐Aldrich) or anti‐laminin (#NB300‐144, Novusbio, Shanghai, China), or anti‐p16 (#PA5‐20379, Invitrogen) or anti‐FOXO3a antibodies (#ab23683, Abcam) in muscle transverse sections overnight at 4°C. After washed three times with 0.1% PBS with Tween 20 (PBST), these sections were incubated with FTIC‐conjugated secondary antibodies (#111‐095‐003, Jackson ImmunoResearch Laboratories, West Grove, PA) and Alexa Fluor 647 (#ab150083, Abcam) for 1 h, respectively. Then, the slides were washed three times with PBST and stained with 1 μg/mL of 4,6‐diamidino‐2‐phenylindole (DAPI) for 30 min to detect nuclei. Images were acquired using a Zeiss Axioskop 2 plus fluorescence microscope. ImageJ software was used to quantify the capillaries, the capillary‐to‐fibre ratio and the expression of *p16*.

### Lifespan assays

All lifespan assays were carried out on NGM agar plates seeded with *E. coli* OP50. The exercised or unexercised 6‐day‐old worms were transferred to fresh plates every other day, and survival was scored until all worms were dead. If worms did not respond to touches with a platinum pick, they were considered as dead.

### Age‐related phenotypic marker assays in worms

The pumping rate was measured by counting the number of contractions in the terminal bulb of pharynx per 30 s. A body bend was defined as a change in direction of midbody bend followed by a return to the original location.

### DAF‐16::GFP localization assay

After swim training, worms were quickly mounted in M9 onto microscope slides. The slides were viewed with a Zeiss Axioskop 2 plus fluorescence microscope. The states of DAF‐16::GFP distribution were categorized as nuclear, intermediate and cytosolic. The status of DAF‐16::GFP distribution was categorized as cytosolic localization or nuclear localization when distribution was observed throughout the body from head to tail. When the nuclear localization of DAF‐16::GFP was visible, but not completely throughout the body, the status of DAF‐16 localization was categorized as intermediate.

### Measurement of collagen content

Total collagen content was determined by measuring the hydroxyproline (HYP) levels.^16^ After worms were homogenized in liquid nitrogen, the resulting powders were acid digested in 0.5 mL of 6‐M HCl at 110°C for 2–6 h. Once the solution became clear, the pH was adjusted to 6–8 by NaOH. The contents of collagen were determined using the HYP Content Assay Kit (BC0250, Solarbio, Beijing, China). The collagen contents were expressed as micrograms of HYP per milligram of worm wet weight.

### Autophagy analysis

The transgenic worms carrying GFP::LGG‐1 were mounted onto microscope slides. The slides were viewed using a Zeiss Axioskop 2 plus fluorescence microscope. The GFP‐positive puncta were counted in the seam cells and intestine.

### Statistical analysis

Differences in survival rates were analysed using the log‐rank test. Differences in gene expression, mRNA and protein levels, total collagen content, age‐related phenotypic marker assays, the numbers of GFP::LGG‐1‐positive puncta and fluorescence intensity were assessed by performing one‐way analysis of variance (ANOVA) followed by a Student–Newman–Keuls test or Student's *t* test. Differences in distribution of DAF‐16 were analysed using the Friedman test (with Dunn's test for multiple comparisons).

## Results

### Activation of the PAQR‐1/AdipoR1 pathway is involved in lifespan extension in swim‐exercised worms

**Figure 1:**
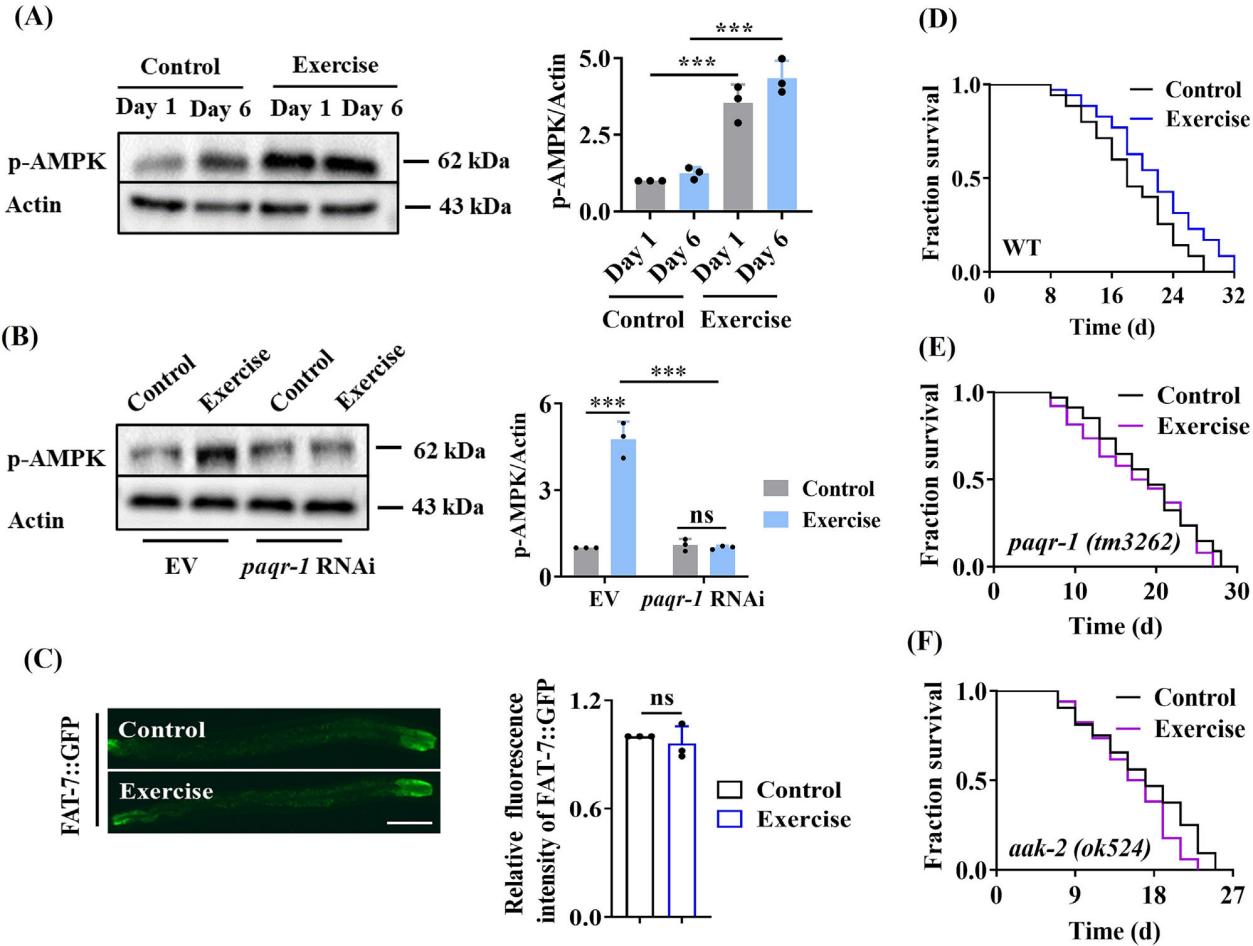
The PAQR‐1/AdipoR1 pathway is involved in lifespan extension in swim‐exercised worms. (A) AMPK was activated by swim exercise in worms. The phosphorylation of AMPK (Thr172) was measured by western blotting (left panel). Quantification of the ratio of p‐AMPK to actin (right panel). (B) Knockdown of *paqr‐1* by RNAi significantly reduced the phosphorylation of AMPK (left panel). Quantification of the ratio of p‐AMPK to actin (right panel). (C) The expression of *fat‐7::gfp* was not altered in swim‐exercised worms. Representative images of FAT::GFP in Day 0 worms after swim exercise (left panel). Quantification of GFP levels (right panel). Scale bars: 100 μm. (D) Swim exercise significantly extended the lifespan in worms. Swim exercise failed to extend the lifespan in *paqr‐1(tm3262)* (E) and *aak‐2(ok524)* (F) mutants. ^***^ *P* < 0.001, swim‐exercised worms versus control worms. ns, not significant.

In *C. elegans* , *paqr‐1* and *paqr‐2* are homologues of the mammalian AdipoR1 and AdipoR2 genes.^16^ To determine whether PAQR‐1 and PAQR‐2 were involved in exercise‐induced longevity in *C. elegans* , we used a swim exercise regimen for worms (90 min/day, *Figure* *S2*).^5^ To test the activation of PAQR‐1, we determined the phosphorylation of AMPK. We found that the levels of phospho‐AMPK (Thr172) were significantly increased in either 1‐day‐old or 6‐day‐old swim‐exercised worms (*Figure* *1A*). Moreover, knockdown of *paqr‐1* by RNAi abolished the increase in phospho‐AMPK in swim‐exercised worms (*Figure* *1B*). In *C. elegans* , *fat‐7* encoding a stearic CoA desaturase is a target of NHR‐49/PPARα.^16^ We found that the expression of *fat‐7p::gfp* was not altered in response to exercise (*Figure* *1C*). These results suggest that swim exercise activates the PAQR‐1 signalling, rather than the PAQR‐2 signalling, in worms.

Consistent with a previous observation,^5^ we found that swim exercise induced lifespan extension in worms (*Figure* *1D*). However, mutations in *paqr‐1* and *aak‐2* encoding the *C. elegans* AMPK α2 catalytic subunit failed to extend the lifespan in swim‐exercised worms (*Figure* *1E,F*). In contrast, exercise still extended the lifespan in *paqr‐2(tm3410)* mutants (*Figure* *S3*). Thus, exercise extends the lifespan in worms by activating the PAQR‐1 signalling.

### Wheel running exercise improves muscle quality via AdipoR1 in aged mice

**Figure 2:**
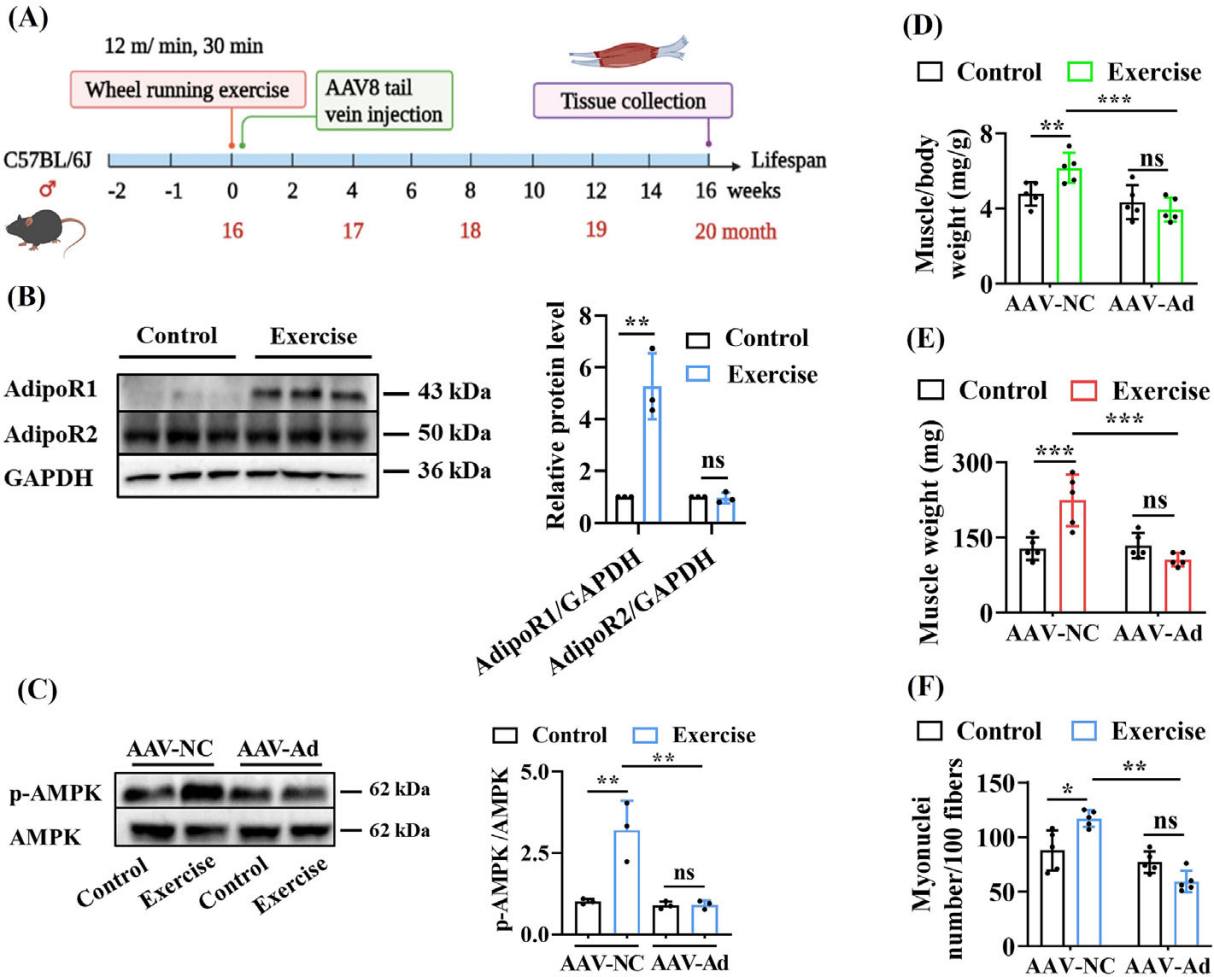
Exercise improves muscle quality via AdipoR1 in mice. (A) Exercise paradigm in mice. Mice at 16 months of age in exercised groups were performed voluntary wheel running for 4 months. (B) The protein levels of AdipoR1 in skeletal muscle were upregulated by exercise. The protein levels of AdipoR1 and AdipoR2 were measured by western blotting (left panel). Quantification of the ratio of either AdipoR1 or AdipoR2 to GAPDH (right panel). (C) Knockdown of Adipor1 inhibited the activation of AMPK induced by exercise. The phosphorylation of AMPK (Thr172) was measured by western blotting (left panel). Quantification of the ratio of p‐AMPK to AMPK (right panel). (D) Muscle mass index of gastrocnemius muscles (*n* = 5 per group). (E) Muscle weight in gastrocnemius muscles of mice (*n* = 5 per group). (F) Myonuclei number in gastrocnemius muscles of mice (*n* = 5 per group). *P < 0.05, ^**^ *P* < 0.01 and ^***^ *P* < 0.001. Ad, AdipoR1; NC, negative control; ns, not significant.

To test the role of the AdipoR1 signalling in improvement of muscle quality by exercise in rodents, aged (16‐month‐old) mice were subjected to a 4‐month wheel exercise training paradigm (*Figure* *2A*). There are three different exercise training modalities (treadmill running, wheel running and swimming) that have been used for training mice. In this study, we chose the motorized running wheel as a training model. In contrast to voluntary wheel running, forced wheel running allows for better control of exercise intensity, time and duration in mice.^22^ We first determined whether exercise training activated the AdipoR1 signalling. We found that exercise increased the protein levels of adiponectin in serum (*Table* *S2*). Moreover, an increase in the protein levels of AdipoR1, rather than AdipoR2, was observed in the skeletal muscle of exercised mice (*Figure* *2B*). The adaptor proteins APPL1 and APPL2 positively and negatively mediate the adiponectin signalling by binding directly with adiponectin receptors, respectively.^23^ We found that exercise increased the protein levels of APPL1, rather than APPL2 (*Figure* *S4A,B*). In addition, exercise increased the phospho‐AMPK (Thr172) levels in the skeletal muscle of aged mice (*Figure* *2C*). Finally, knockdown of *AdipoR1* by shRNA abolished the increase in the levels of phospho‐AMPK (*Figure* *2C*). These results suggest that exercise activates the AdipoR1 signalling in mice. To investigate the upstream of the signalling pathways for AMPK activation during exercise, we determined the phosphorylation levels of LKB1 and CaMKKβ, both of which can phosphorylate AMPK.^24^, ^25^ However, exercise did not affect the levels of phospho‐LKB1 and phospho‐CaMKKβ (*Figure* *S4C,D*). Importantly, knockdown of *AdipoR1* did not affect the levels of phospho‐LKB1 and phospho‐CaMKKβ in exercised mice (*Figure* *S4C,D*).

Next, we found that exercise significantly increased muscle mass index (*Figure* *2D*) and muscle weight in mice (*Figure* *2E*). Histopathologically, muscle atrophy is characterized by a reduction in myofibres, myonuclei and myofibre CSA. Using HE staining, we found that 20‐month‐old mice exhibited marked muscle atrophy, compared to 16‐month‐old mice (*Figure* *S5A* and *Table* *S3*). In contrast, the myofibres were closely arranged, and the gaps between muscle fibres were narrowed in 20‐month‐old exercised mice (*Figure* *S5A,B*). Furthermore, exercise increased muscle fibre CSA (*Figure* *S5C*) and myonuclei number of gastrocnemius muscle (*Figure* *2F*). Knockdown of *AdipoR1* by RNAi abrogated the improvement in muscle quality by exercise (*Figures* *2D*–*F* and *S5B,C* and *Table* *S2*).

The protein and mRNA levels of p16^INK4a^, a marker for cellular senescence, were markedly reduced in skeletal muscle of exercised mice (*Figure* *S6A–C*). However, the p16^INK4a^ protein and mRNA levels were upregulated in exercised mice treated with *AdipoR1* shRNA, compared with those subjected to scrambled shRNA (*Figure* *S6A–C*). Furthermore, blood flow to skeletal muscle is an important feature in the reduction of skeletal muscle function with ageing. We thus examined the abundance of capillaries in skeletal muscle by detecting CD31 (capillaries) and laminin (muscle stroma). The number and density of capillaries in the gastrocnemius muscle of exercised mice were significantly higher than those in the control mice (*Figure* *S7A–D*). However, knockdown of *AdipoR1* significantly abolished this increase in the number and density of capillaries (*Figure* *S7A–D*). These results suggest that exercise improves skeletal muscle quality and reduces muscle cell senescence and capillary density via AdipoR1.

### FOXO3a functions downstream of AdipoR1 to prevent skeletal muscle loss via autophagy

**Figure 3:**
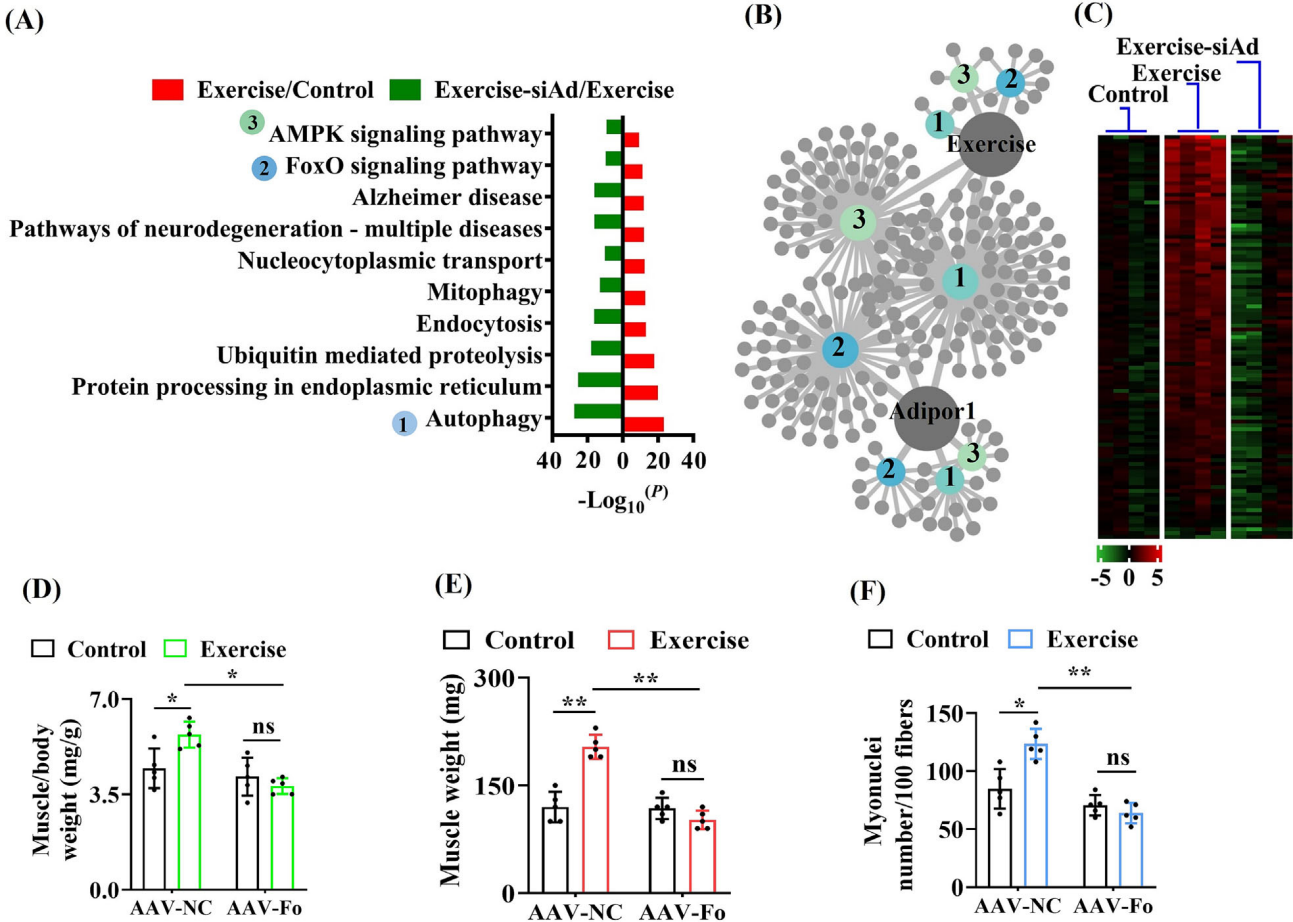
FOXO3a functions as a downstream molecule of AdipoR1 and improves muscle quality induced by exercise in mice. (A) Enrichment analysis of KEGG pathways was identified using the DAVID annotation tool. (B) Genes related to the FOXO signalling pathway, autophagy and the AMPK signalling pathway were regulated by AdipoR1, and exercise is shown in grey circles. 1, autophagy; 2, the FOXO signalling pathway; 3, the AMPK signalling pathway. (C) AdipoR1 regulated FOXO3a‐dependent genes. (D) Muscle mass index of gastrocnemius muscles (*n* = 5 per group). (E) Muscle weight in gastrocnemius muscles of mice (*n* = 5 per group). (F) Myonuclei number in gastrocnemius muscles of mice (*n* = 5 per group). *P < 0.05 and ^**^ *P* < 0.01. Fo, FoxO3a; NC, negative control; ns, not significant; siAd, knockdown of AdipoR1 by RNAi.

To identify the mechanism underlying AdipoR1‐mediated beneficial effect of exercise, we used RNA‐Seq analysis to study the transcriptomic profiles of skeletal muscle in mice after exercise. Among the differentially expressed genes (DEGs) in exercised mice, 6539 genes were upregulated and 107 downregulated (*Figure* *S8A* and *Table* *S4*). In exercised mice subjected to *AdipoR1* shRNA, 109 genes were upregulated and 7407 downregulated (*Figure* *S8B* and *Table* *S5*). There were 10 KEGG pathways overrepresented in the upregulated genes in exercised mice and in the downregulated genes in exercised mice with knockdown of *AdipoR1* (*Figure* *3A*). These overlapped categories represent the pathways that are activated by exercise in an AdipoR1‐dependent manner, including ‘AMPK signalling’, ‘FOXO signalling’, ‘autophagy’ and ‘mitophagy’ (*Figure* *3A,B*).

In this study, most of upregulated genes in ‘FOXO signalling pathway’ in exercised mice were downregulated by knockdown of *AdipoR1* (*Figure* *3C*). FOXO3a is a downstream molecule of the AdipoR1 signalling.^13^ However, we found that FOXO3a was predominantly located in the nucleus, which was not affected by either exercise or AdipoR1 knockdown (*Figure* *S9*). Thus, the AdipoR1 signalling promotes the transcriptional activity of FOXO3a, rather than nuclear translocation, in exercised mice. Next, knockdown of *FoxO3a* by shRNA suppressed the increase in muscle mass index (*Figure* *3D* and *Table* *S6*), muscle weight (*Figure* *3E* and *Table* *S6*), muscle fibre CSA (*Figure* *S5D*) and myonuclei number of gastrocnemius muscle in exercised mice (*Figure* *3F*). Furthermore, the p16^INK4a^ protein and mRNA levels were increased in exercised mice treated with *FoxO3a* shRNA (*Figure* *S10A–C*). Meanwhile, knockdown of *FoxO3a* significantly abolished this increase in the abundance of capillaries (*Figure* *S11A–D*).

**Figure 4:**
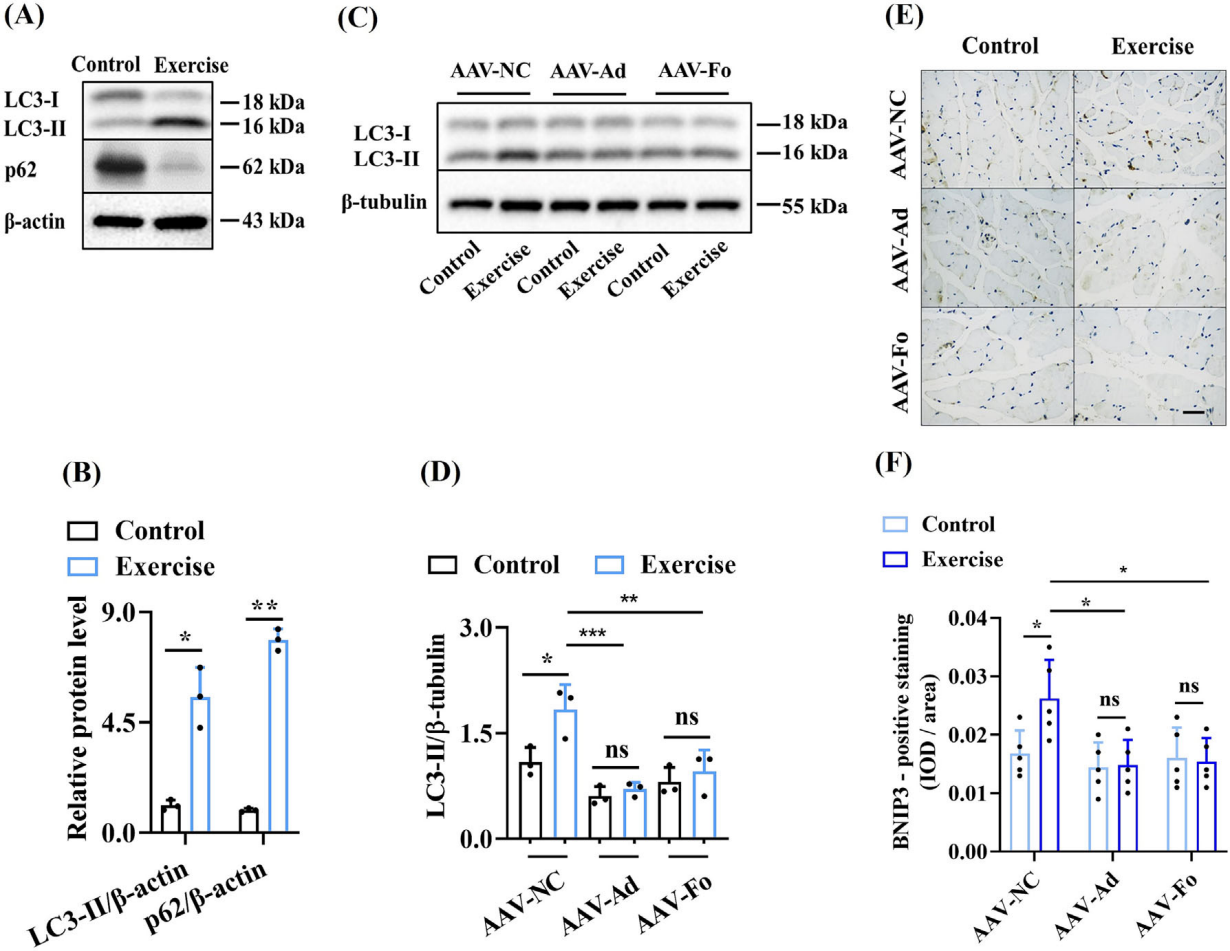
The AdipoR1–FOXO3a signalling induces autophagy in skeletal muscle of mice after exercise. (A, B) Exercise significantly increased the levels of the LC3B‐II and reduced the levels of p62 in skeletal muscle of exercised mice. The blot is typical of three independent experiments (A). Quantification of the ratio of LC3B‐II to β‐actin (B). Quantification of the ratio of p62 to β‐actin (B). (C, D) Knockdown of either *AdipoR1* or *FoxO3a* by shRNA reduced the levels of the LC3B‐II in skeletal muscle of exercised mice. The blot is typical of three independent experiments (C). Quantification of the ratio of LC3B‐II to β‐tubulin (D). (E, F) Exercise upregulated the protein expression of BNIP3 in the skeletal muscle of mice. Knockdown of either *AdipoR1* or *FoxO3a* by shRNA reduced the levels of the BNIP3 in the skeletal muscle of exercised mice. Representative images of immunohistochemical staining for BNIP3 (E). Scale bars: 50 μm. Quantification of BNIP3 expression in gastrocnemius muscles of mice (F). *P < 0.05, ^**^ *P* < 0.01 and ^***^ *P* < 0.001. Ad, AdipoR1; Fo, FoxO3a; NC, negative control; ns, not significant.

Exercise training activates autophagy/mitophagy in skeletal muscle of mice.^7^ We also observed that ‘autophagy’ and ‘mitophagy’ were two of these overrepresented categories (*Figure* *3A*). Exercise significantly increased the levels of the endogenous lapidated LC3B‐II form by western blotting (*Figure* *4A,B*). The increase in LC3B‐II by exercise could result from either an induction of autophagy or a block in the turnover of LC3B‐II‐bound autophagosomes. For instance, administration with colchicine, an inhibitor of autophagosome–lysosome fusion that directly blocks autophagy flux in skeletal muscle, significantly increases the proteins levels of LC3‐II and p62 in skeletal muscle of exercise mice.^26^ To distinguish between these possibilities, we detected the turnover of p62/SQSTM1. We found that exercise significantly reduced the levels of p62/SQSTM1 in skeletal muscle of exercised mice (*Figure* *4A,B*). Meanwhile, we also detected the mRNA levels of autophagy/mitophagy‐related genes (*Becn1*, *Ulk1*, *Pik3c3*, *Atg5*, *Atg7*, *Lc3b*, *Bnip3* and *Pink1*) and found that exercise upregulated the expressions of these genes (*Figure* *S12*). Furthermore, knockdown of either *AdipoR1* or *FoxO3a* suppressed the protein levels of LC3‐II (*Figure* *4C,D*) and BNIP3 (*Figure* *4E,F*), an important mediator of mitophagy, in the skeletal muscle of exercised mice. These results indicate that exercise induces autophagy/mitophagy via AdipoR1 and FOXO3a in the skeletal muscle of mice.

### Increased DAF‐16 transcription activity by the PAQR‐1 signalling extends the lifespan in swim‐exercised worms

**Figure 5:**
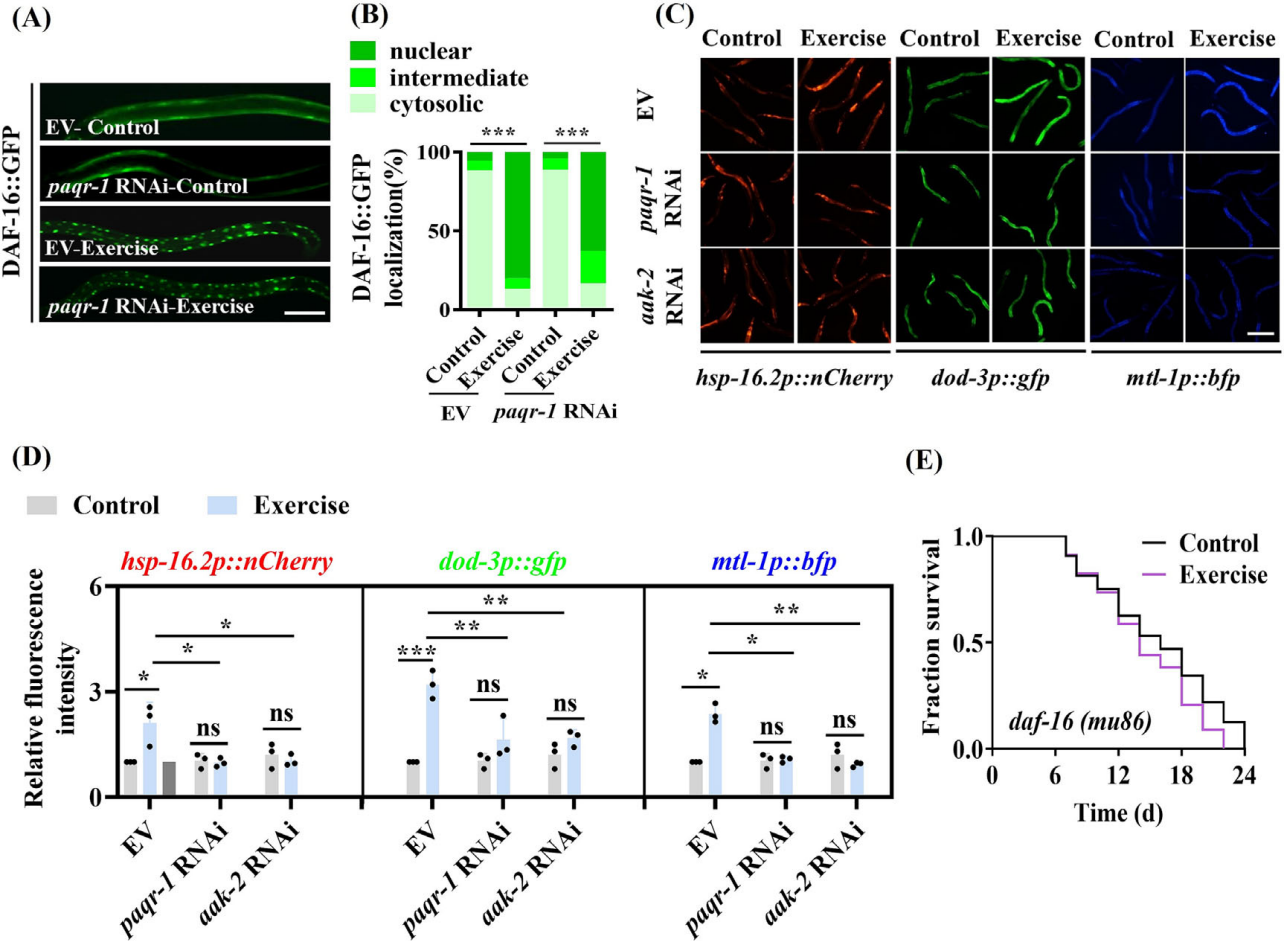
DAF‐16 is involved in lifespan extension in swim‐exercised worms. (A, B) Swim exercise significantly increased the nuclear accumulation of DAF‐16::GFP. Knockdown of *paqr‐1* by RNAi did not affect the nuclear localization of DAF‐16 induced by swim exercise. Representative images of DAF‐16::GFP expression pattern in worms (A). Scale bars: 100 μm. Quantification of DAF‐16 distribution (B). (C, D) Swim exercise upregulated the expressions of *hsp‐16.2::nCherry*, *dod‐3::gfp* and *mtl‐1p::bfp*. However, RNAi knockdown of *paqr‐1* or *aak‐2* reduced the expressions of these three genes. Representative images of *dod‐3p::gfp*, *hsp‐16.2p::nCherry* and *mtl‐1p::bfp* in worms after swim exercise (C). Scale bars: 200 μm. Quantification of fluorescent intensity of *hsp‐16.2p::nCherry*, *dod‐3p::gfp* and *mtl‐1p::bfp* (D) in worms. (E) Swim exercise failed to extend the lifespan in *daf‐16(mu86)* worms. These results are means ± SD of three independent experiments (*n* = 30 worms per experiment). *P < 0.05, ^**^ *P* < 0.01 and ^***^ *P* < 0.001. ns, not significant.

To test whether the PAQR‐1 signalling regulates DAF‐16/FOXO in worms, we first monitored the nuclear translocation of DAF‐16, which is an indicator of its activation, using transgenic worms expressing *daf‐16p::daf‐16::gfp*. Swim exercise significantly increased the nuclear accumulation of DAF‐16::GFP (*Figure* *5A,B*). Intriguingly, RNAi knockdown of *paqr‐1* or *aak‐2* did not affect its nuclear localization by exercise (*Figures* *5A,B* and *S13A,B*). As the activity of DAF‐16 is not only decided by its nuclear translocation,^27^ we assayed DAF‐16 target genes, such as *hsp‐16.2*, *dod‐3* and *mtl‐1*. We found that swim exercise significantly promoted the expressions of *hsp‐16.2::nCherry*, *dod‐3::gfp* and *mtl‐1p::bfp* (*Figure* *5C,D*). However, silencing of *paqr‐1* or *aak‐2* by RNAi led to a significant reduction in the expressions of these genes (*Figure* *5C,D*). Furthermore, a mutation in *daf‐16(mu86)* abolished exercise‐induced lifespan extension in worms (*Figure* *5E*). These data indicate that the activation of DAF‐16 is required for exercise‐mediated lifespan extension in worms.

### DAF‐16 promotes autophagy/mitophagy, which is required for lifespan extension in swim‐exercised worms

**Figure 6:**
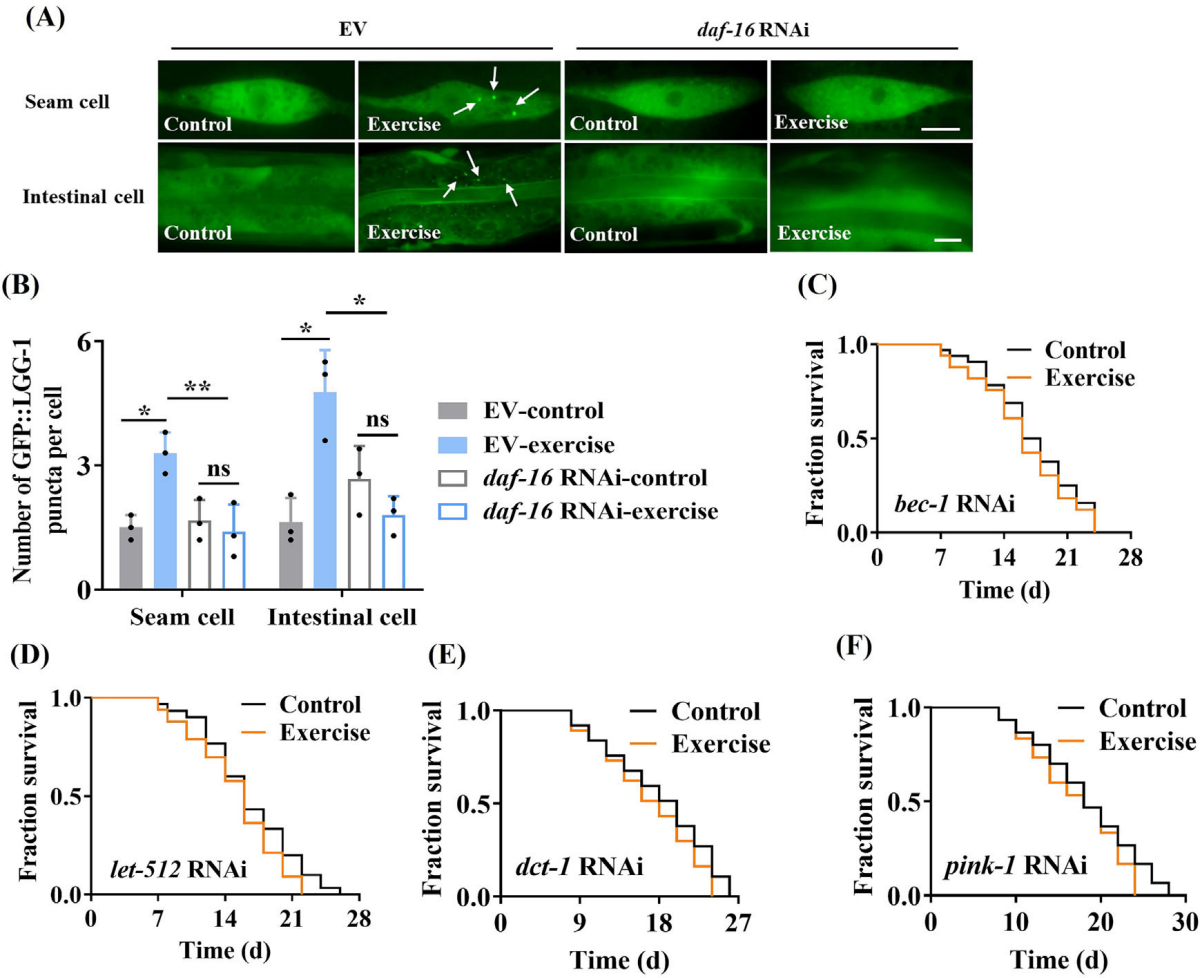
DAF‐16 induces autophagy, which is required for lifespan extension in swim‐exercised worms. (A) Representative images of autophagosomes (GFP::LGG‐1 puncta) in the seam cells and intestine of 0‐day‐old worms subjected to swim exercise. The arrow denotes a representative autophagosome. Knockdown of DAF‐16 by RNAi reduced autophagy induced by swim exercise, which was detected by GFP::LGG‐1 puncta in the seam cells and intestine of swim‐exercised worms. Scale bars: 10 μm. (B) The numbers of GFP::LGG‐1 puncta were counted. These results are means ± SD of three independent experiments (*n* = 30–35 worms per experiment). (C, D) Autophagy was required for lifespan extension in swim‐exercised worms. Swim exercise failed to extend the lifespan in worms subjected to *bec‐1* (C) or *unc‐51* RNAi (D). (E, F) Mitophagy was involved in lifespan extension in swim‐exercised worms. Swim exercise failed to extend the lifespan in worms subjected to *dct‐1* (E) or *pink‐1* RNAi (F). *P < 0.05 and ^**^ *P* < 0.01. ns, not significant.

To test whether DAF‐16 regulated autophagy in swim‐exercised worms, we measured autophagy levels by using transgenic worms carrying GFP::LGG‐1. We observed that exercise significantly increased autophagy in worms, as evidenced by increased GFP::LGG‐1‐positive puncta in the seam cells and intestine of worms^28^ (*Figure* *6A,B*). Swim exercise also resulted in a significant increase in the ratio of phosphatidylethanolamine (PE)‐conjugated GFP::LGG‐1 (PE‐GFP::LGG‐1) to non‐lipidated GFP::LGG‐1 in 1‐day‐old and 6‐day‐old worms (*Figure* *S14A,B*). Knockdown of *daf‐16* by RNAi prominently reduced autophagy (*Figure* *6A,B*). Similar results were obtained in swim‐exercised worms subjected to *paqr‐1* RNAi (*Figure* *S15A–D*).

Next, we found that swim exercise failed to extend the lifespan in worms subjected to RNAi of autophagic genes, including *bec‐1*, *let‐512*, *epg‐1* and *unc‐51* (*Figures* *6C,D* and *S16A,B*). Mitophagy is a major type of selective autophagy that specifically degrades dysfunctional mitochondria.^14^ We found that the mRNA levels of *pink‐1* ( *C. elegans* homologue of BCL2/adenovirus E1B‐interacting protein 3) and *dct‐1* ( *C. elegans* homologue of mitochondrial PTEN‐induced kinase 1) were decreased in swim‐exercised worms subjected to *daf‐16* RNAi (*Figure* *S17A,B*). Finally, exercise no longer extended the lifespan in worms subjected to either *dct‐1* or *pink‐1* RNAi (*Figure* *6E,F*). Thus, the activation of DAF‐16 is required for exercise‐mediated lifespan extension via autophagy/mitophagy in worms.

### Autophagy/mitophagy is required for exercise‐induced healthy longevity in worms

**Figure 7:**
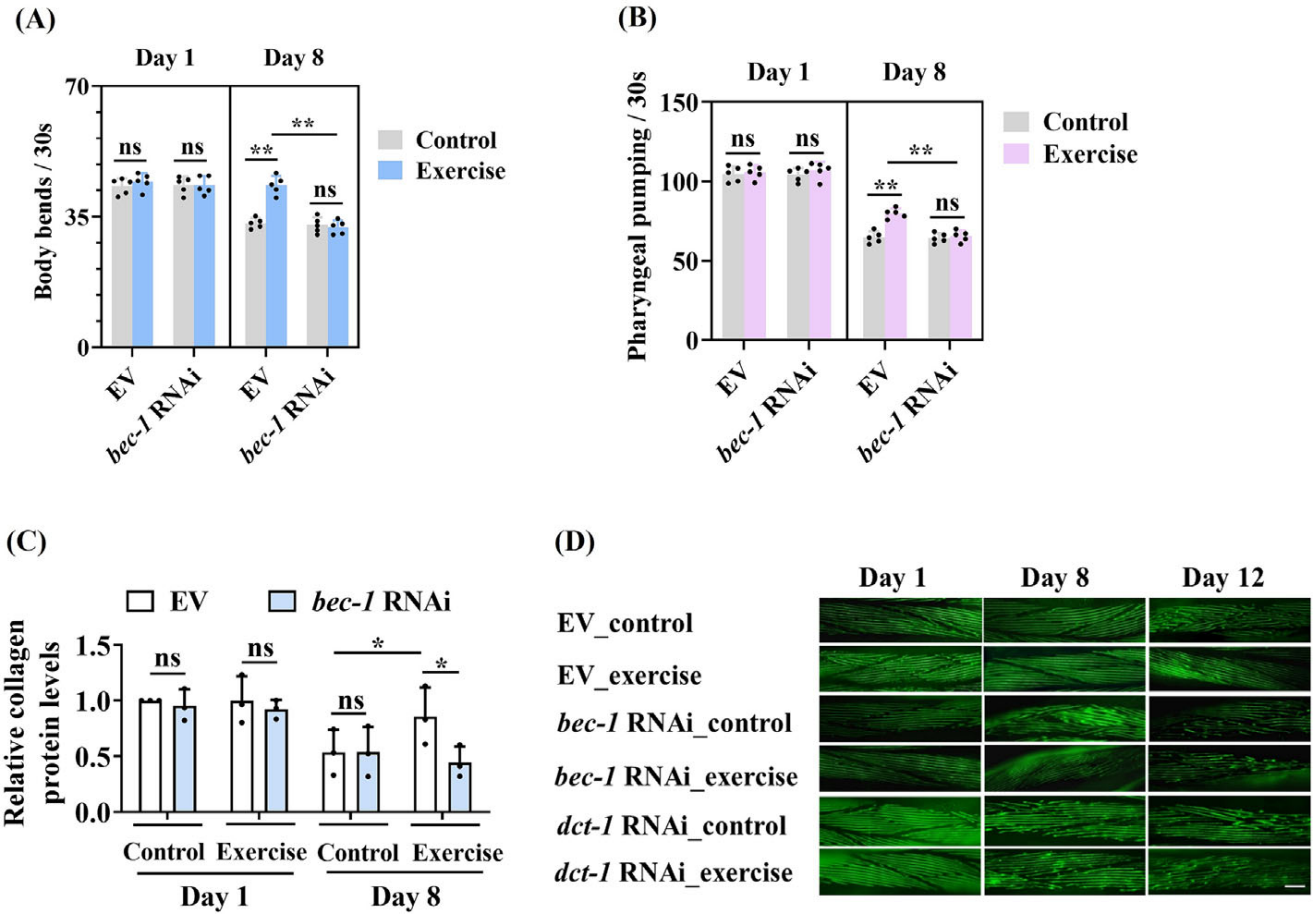
Autophagy/mitophagy is required for exercise‐induced healthy longevity in worms. Autophagy was involved in delaying the appearance of the ageing markers, including body bending (A), pharyngeal pumping (B) and relative collagen levels (C), in swim‐exercised worms. These results are means ± SD of three or five independent experiments (*n* = 30–35 worms per experiment). (D) Swim exercise restored age‐related muscle deterioration in an autophagy‐dependent manner. Representative images of *myo‐3p::GFP::myo‐3* in exercised worms. Scale bars: 10 μm. *P < 0.05 and ^**^ *P* < 0.01. ns, not significant.

We determined the role of the PAQR‐1/AdipoR1 pathway in phenotypic traits, such as body bending, pharyngeal pumping rate and collagen contents, which are reduced with ageing in worms. Swim exercise resulted in an increase in the rates of body bending (*Figure* *7A*), pharyngeal pumping (*Figure* *7B*) and collagen contents (*Figure* *7C*) in 8‐day‐old worms. However, knockdown of *bec‐1*, *dct‐1*, *unc‐51* or *pink‐1* by RNAi abolished the beneficial effect of swim exercise on these phenotypic traits (*Figures* *7A–C* and *S18A–I*). In addition, we observed age‐related deterioration of body wall muscle by using transgenic worms carrying *myo‐3p::GFP::myo‐3*. The muscle fibres were disrupted and arranged sparsely in 12‐day‐old control worms (*Figure* *7D*). The muscle fibres exhibited more organized in 12‐day‐old swim‐exercised worms, compared with those in age‐matched control worms. However, either *bec‐1* or *dct‐1* RNAi blocked the beneficial effect of exercise on body wall muscle of 12‐day‐old worms (*Figure* *7D*). These results suggest that autophagy/mitophagy is sufficient for exercise‐induced healthy longevity in worms.

## Discussion

In this study, our data demonstrate that the PAQR‐1/AdipoR1 signalling, but not the PAQR‐2/AdipoR2 signalling, is involved in lifespan extension in swim‐exercised worms. Furthermore, long‐term exercise alleviates age‐related skeletal muscle decline via the AdipoR1 signalling in mice. Our findings reveal that the AdipoR1 signalling acts as a critical regulator of exercise‐induced healthspan and suggest that such a mechanism may be evolutionally conserved in diverse organisms.

Our results indicate that *C. elegans* PAQR‐1/AdipoR1 regulates the phosphorylation of AMPK, whereas PAQR‐2/AdipoR2 controls the transcription activity of NHR‐49/PPARα in worms.^16^ In *Drosophila*, dAdipoR (an orthologue of mammalian AdipoR1) also controls glucose and lipid metabolism.^29^ These results suggest that the AdipoR signalling is conserved across species. It should be noted that no obvious homologue of adiponectin exists in worm and fly genomes.^29^, ^30^ How to activate the PAQR‐1/AdipoR1 signalling by exercise in these species remains inconclusive. In humans and rodents, exercise increases the adiponectin levels in serum^7^ and upregulates the AdipoR1 expression in skeletal muscle.^31^ Our results not only are consistent with these observations but also demonstrate that exercise upregulates the expression of APPL1, the adaptor protein that positively mediates adiponectin signalling.^23^ Thus, our results support the idea that APPL1 is likely involved in activation of the adiponectin signalling during exercise.^32^

AMPK plays an important role in exercise‐induced autophagy in skeletal muscle of rodents.^14^ LKB1 and CaMKKβ are two upstream kinases for AMPK activation in skeletal muscle during contraction.^33^ Knockdown of either CaMKKβ or LKB1 slightly but significantly reduces the increases in phospho‐AMPK (Thr172) induced by adiponectin.^15^ These data raise a possibility that these two upstream kinases are involved in exercise‐mediated AMPK activation via AdipoR1. However, our data demonstrate that exercise does not activate both LKB1 and CaMKKβ. Likewise, knockdown of *AdipoR1* does not influence the activities of phospho‐LKB1 and phospho‐CaMKKβ in exercised mice. Thus, the causal roles for these two upstream kinases‐mediated AMPK activation by exercise are excluded. Our results reveal that DAF‐16/FOXO3a functions downstream of the PAQR‐1/AdipoR1‐AMPK signalling to promote autophagy by exercise. Notably, PAQR‐1 and AdipoR1 promote exercise‐mediated transcriptional activation of DAF‐16 and FOXO3a, but do not affect their nuclear localization in both worms and mice. These results support the notion that AMPK phosphorylates and activates DAF‐16 and FOXO3a without affecting their subcellular localization in worms and mice, respectively.^13^, ^34^ As pharmacological activation of AMPK alone is not sufficient to induce autophagy in skeletal muscle of mice,^35^ additional mechanisms must contribute to FOXO3a‐induced autophagy during exercise.

FOXO3a plays a guardian role in maintaining tissue homeostasis in the process of ageing.^36^ Our results suggest that FOXO3a prevents age‐related muscle atrophy by exercise via induction of autophagy. However, activation of autophagy via FOXO3a stimulates the loss of muscle protein in muscles atrophying due to fasting or denervation.^11^ The most parsimonious explanation for these conflicting observations is that the role of FOXO‐mediated autophagy in muscle homeostasis is more complex and context dependent. Indeed, too much autophagy under pathological events causes excessive removal of cellular components and leads to the atrophy of skeletal muscle.^37^ In contrast, the appropriate induction of autophagy is required for the clearance of aggregated proteins and the homeostatic maintenance of skeletal muscle in normal ageing process.^38^

In conclusion, our finding reveals an evolutionarily conserved pathway by which activation of the PAQR‐1/AdipoR1 signalling by exercise delays age‐associated skeletal muscle decline in mice and promotes longevity in worms via DAF‐16/FOXO3a‐induced autophagy. Our data also support the view that *C. elegans* is a promising model for understanding the beneficial effects of exercise.^5^ Recent studies identified several signalling pathways as modulators of the benefits of exercise.^39^, ^40^ For instance, irisin, the cleaved and circulating form of fibronectin‐domain III containing 5, confers the beneficial effects of exercise on browning of adipose tissues and cognitive function in mice.^39^ Uncovering the mechanisms by which exercise regulates healthspan may provide novel therapeutic strategies to alleviate aged‐related diseases.

## Conflict of interest statement

The authors declare no conflicts of interest.

## Funding

This work was supported by grants from National Natural Science Foundation of China (82201726) and State Key Laboratory for Conservation and Utilization of Bio‐Resources in Yunnan (2021KF001), and Joint Funds for Department of Science and Technology of Yunnan‐Kunming Medical University (202001AY070001‐013).

## Supporting information


**Figure S1.** The regions of the gastrocnemius muscle used for each assay. WB, Western blotting; IHC, Immunohistochemical analysis; IF, immunofluorescence staining. H&E, Haematoxylin and eosin (HE) staining.
**Figure S2.** Swim exercise regimen for the nematode *C. elegans* . Starting at the L4 larvae stage, exercise for 90 minutes once a day for eight days. The day when eggs were laid was recorded as day 1 of adulthood.
**Figure S3.** *paqr‐2* is not required for exercise‐mediated lifespan extension. Swim exercise still extended the lifespan in *paqr‐2*(*tm3410*) mutants. *P < 0.05, swim‐exercised worms versus control worms.
**Figure S4.** The effect of exercise on the major molecules in the AdipoR1‐AMPK signaling. (A and B) Physical exercise up‐regulated the protein levels of APPL1, rather than APPL2, in skeletal muscle of mice. The protein levels of APPL1 (A) and APPL2 (B) were measured by Western blotting (left panel). Quantification of the ratio of APPL1 (A) and APPL2 (B) to GAPDH (right panel). (C and D) Physical exercise did not affect the phosphorylation of LKB1 and CaMKKβ in skeletal muscle of mice. Furthermore, knockdown of AdipoR1 by RNAi did not affect the phosphorylation of LKB1 and CaMKKβ. The levels of phospho‐LKB1(Ser428) (C) and phospho‐CaMKKβ(Ser511) (D) were measured by Western blotting (left panel). Quantification of the ratio of p‐LKB1 to LKB1 (C) or p‐CaMKKβ to CaMKKβ (D) (right panel). *P < 0.05. ns, not significant. NC, negative control. Ad, AdipoR1.
**Figure S5.** Exercise improves muscle quality, which is dependent on AdipoR1 and FoxO3a (A) Representative haematoxylin and eosin (HE) staining in transverse gastrocnemius muscles sections from mice of 16‐month‐old and 20‐month‐old. Scale bar, 50 μm. (B) Representative haematoxylin and eosin (HE) staining in transverse gastrocnemius muscle sections of 20‐month‐old mice. Scale bar, 100 μm. (C and D) Muscle fiber cross‐sectional area (CSA) in gastrocnemius muscles of mice (*n* = 5 per group). *P < 0.05, **P < 0.01, ***P < 0.001, ns, not significant. NC, negative control. Ad, AdipoR1. Fo, FoxO3a
**Figure S6.** Exercise reduces the expression of p16INK4a via AdipoR1 in skeletal muscle of mice. (A and B) The protein levels of p16^INK4a^ were reduced in skeletal muscle of exercised mice. This decrease was inhibited by knockdown of AdipoR1 by shRNA. Representative images of immunofluorescence staining for p16^INK4a^ in gastrocnemius muscles of mice. Scale bar, 20 μm (A). Quantification of p16Ink4a protein expression (B). (C) Quantitative real‐time PCR analysis of *p16* ^*Ink4a*^ mRNA expression. **P < 0.01, ***P < 0.01, ns, not significant. NC, negative control. Ad, AdipoR1.
**Figure S7.** AdipoR1 is involved in the exercise‐induced improvement of muscle atrophy. (A) Representative images of CD31 and Laminin immunofluorescence staining in mice gastrocnemius muscles. NC, negative control. Ad, AdipoR1. Scale bar, 40 μm. (B‐D) Exercise increased the number of capillaries (B), the ratio of capillary to fiber (C), and fiber cross‐sectional area (CSA) (D) in the gastrocnemius muscles. These increases were inhibited by knockdown of AdipoR1 by shRNA. Values are presented as mean ± SD, *n* = 5 per group. *P < 0.05, **P < 0.01, ***P < 0.01, ns, not significant. NC, negative control. Ad, AdipoR1.
**Figure S8.** RNA‐Seq analysis the transcriptomic profiles of mice after wheel running exercise. (A and B) Volcano Plot for differential gene expression was performed by comparing exercised mice to control mice (A), and exercised mice subjected to AdipoR1 shRNA versus exercised mice (B). The red dots represent the significantly upregulated genes and the green dots represent the significantly downregulated genes.
**Figure S9.** Subcellular localization of FOXO3a in skeletal muscle is not affected by either exercise or AdipoR1 knockdown. Representative images of immunofluorescence staining for FOXO3a in gastrocnemius muscles of mice. NC, negative control. Ad, AdipoR1. Scale bar, 100 μm.
**Figure S10.** Exercise reduces the expression of p16INK4a via FOXO3a in skeletal muscle of mice. (A and B) The protein levels of p16^INK4a^ were reduced in skeletal muscle of exercised mice. This decrease was inhibited by knockdown of FoxO3a by shRNA. Representative images of immunofluorescence staining for p16^INK4a^ in gastrocnemius muscles of mice. Scale bar, 20 μm (A). Quantification of p16Ink4a protein expression (B). (C) Quantitative real‐time PCR analysis of *p16* ^*Ink4a*^ mRNA expression. *P < 0.05, **P < 0.01, ns, not significant. NC, negative control. Fo, FoxO3a.
**Figure S11.** Exercise improves number and density of capillaries via FOXO3a in skeletal muscle of mice. (A) Representative images of immunofluorescence staining for CD31 and Laminin in gastrocnemius muscles of mice, Scale bar, 100 μm. (B‐D) Exercise increased the number of capillaries (B), the ratio of capillary to fiber (C), and fiber cross‐sectional area (CSA) (D) in the gastrocnemius muscles. These increases were inhibited by knockdown of FoxO3a by shRNA. Values are presented as mean ± SD, *n* = 5 per group. *P < 0.05, **P < 0.01, ns, not significant. NC, negative control. Fo, FoxO3a.
**Figure S12.** Exercise up‐regulates the expression of these autophagy‐related genes in worms. These results are means ± SD of three independent experiments. *P < 0.05. **P < 0.01,
**Figure S13.** Exercise induces the nuclear accumulation of DAF‐16, which is independent of AMPK in worms. (A and B) Swim exercise significantly increased the nuclear accumulation of DAF‐16::GFP. RNAi knockdown of *aak‐2* encoding the *C. elegans* AMPK α2 catalytic subunit did not affect the nuclear localization of DAF‐16 induced by swim exercise. Representative images of DAF‐16::GFP expression pattern in worms (A). Scale bars: 100 μm. Quantification of DAF‐16 distribution (B). ***P < 0.001.
**Figure S14.** Exercise promotes autophagy in worms. (A) The protein levels of PE‐GFP‐LGG‐1 and GFP‐LGG‐1 in worms were measured by Western blotting. The blot shown here is typical of three independent experiments. (B) Quantification of the ratio of PE‐GFP‐LGG‐1 to GFP‐LGG‐1. These results are means ± SD of three independent experiments. **P < 0.01, ***P < 0.001.
**Figure S15.** PAQR‐1 is involved in autophagy induction in exercised worms. (A) Representative images of autophagosomes (GFP::LGG‐1 puncta) in the seam cells of control worms and swim‐exercised worms, respectively. The arrow denotes a representative autophagosome. Scale bars: 10 μm. (B) The numbers of GFP::LGG‐1 puncta were counted in the seam cells. Values are presented as mean ± SD of three independent experiments (*n* = 30–35 worms per experiment). (C) Representative images of autophagosomes (GFP::LGG‐1 puncta) in the intestinal cells of control worms and swim‐exercised worms, respectively. The arrow denotes a representative autophagosome. Scale bars: 10 μm. (D) The numbers of GFP::LGG‐1 puncta were counted in the intestinal cells. Values are presented as mean ± SD of three independent experiments (*n* = 30–35 worms per experiment). *P < 0.05, exercise group versus control group. ns, not significant.
**Figure S16.** Autophagy‐related genes are involved in exercise‐induced longevity in worms. (A and B) Swim exercise failed to extend lifespan in worms subjected to *epg‐1*(A) or *unc‐51* (B) RNAi, respectively. ns, not significant, exercise group versus control group
**Figure S17.** The mRNA levels of *pink‐1* and *dct‐1* are decreased in swim exercised‐worms subjected to daf‐16 RNAi. (A and B) Quantitative real‐time PCR analysis of *pink‐1*(A) or *dct‐1*(B) mRNA expression of control worms and swim‐exercised worms, respectively. Values are presented as mean ± SD, *n* = 5 per group, *P < 0.05. ns, not significant.
**Figure S18.** Knockdown of *let‐512*, *dct‐1* and *pink‐1* by RNAi abolishes the beneficial effect of exercise on aging biomarkers in worms. (A‐C) *let‐512* was involved in delaying the appearance of the aging markers, including body bending (A) pharyngeal pumping (B) and relative collagen levels (C), in swim‐exercised worms. Values are presented as mean ± SD of three or five independent experiments (*n* = 30–35 worms per experiment). (D‐F) *dct‐1* was involved in delaying the appearance of the aging markers, including body bending (D) pharyngeal pumping (E) and relative collagen levels (F), in swim‐exercised worms. Values are presented as mean ± SD of three or five independent experiments (*n* = 30–35 worms per experiment). (G‐I) *pink‐1* was involved in delaying the appearance of the aging markers, including body bending (G) pharyngeal pumping (H) and relative collagen levels (I), in swim‐exercised worms. Values are presented as mean ± SD of three or five independent experiments (*n* = 30–35 worms per experiment). *P < 0.05, **P < 0.01, ***P < 0.001, ns, not significant.

**Data S1.** Supporting Information

**Table S1:** List of primers used for qRT‐PCR assay.

**Table S2.** Serum adiponectin,blood glucose,body weight,weight of gastrocnemius muscles,muscle/body weight,Epididymal white adipose tissue in AAV‐NC and AAV‐Ad mice

**Table S3.** Body weight,weight of gastrocnemius muscles,muscle/body weight in16‐month‐old mice and 20‐month‐old mice.

**Table S4.** Differential expression analysis was performed by comparing exercised mice to control mice

**Table S5.** Differential expression analysis was performed by comparing exercised mice subjected to AdipoR1 shRNA to exercised mice.

**Table S6.** Serum adiponectin,blood glucose,body weight,weight of gastrocnemius muscles,muscle/body weight,Epididymal white adipose tissue in AAV‐NC and AAV‐Fo mice.
