# Supplementary material for: Physical exercise attenuates age‐related muscle atrophy and exhibits anti‐ageing effects via the adiponectin receptor 1 signalling
Source: J Cachexia Sarcopenia Muscle. 2023 May 24;14(4):1789–801. doi: 10.1002/jcsm.13257 (PMC10401527; doi:10.1002/jcsm.13257)
Supplement: Supplementary file 1 — Figure S1. The regions of the gastrocnemius muscle used for each assay. WB, Western blotting; IHC, Immunohistochemical analysis; IF, immunofluorescence staining. H&E, Haematoxylin and eosin (HE) staining. Figure S2. Swim exercise regimen for the nematode C. elegans . Starting at the L4 larvae stage, exercise for 90 minutes once a day for eight days. The day when eggs were laid was recorded as day 1 of adulthood. Figure S3. paqr‐2 is not required for exercise‐mediated lifespan extension. Swim exercise still extended the lifespan in paqr‐2(tm3410) mutants. *P < 0.05, swim‐exercised worms versus control worms. Figure S4. The effect of exercise on the major molecules in the AdipoR1‐AMPK signaling. (A and B) Physical exercise up‐regulated the protein levels of APPL1, rather than APPL2, in skeletal muscle of mice. The protein levels of APPL1 (A) and APPL2 (B) were measured by Western blotting (left panel). Quantification of the ratio of APPL1 (A) and APPL2 (B) to GAPDH (right panel). (C and D) Physical exercise did not affect the phosphorylation of LKB1 and CaMKKβ in skeletal muscle of mice. Furthermore, knockdown of AdipoR1 by RNAi did not affect the phosphorylation of LKB1 and CaMKKβ. The levels of phospho‐LKB1(Ser428) (C) and phospho‐CaMKKβ(Ser511) (D) were measured by Western blotting (left panel). Quantification of the ratio of p‐LKB1 to LKB1 (C) or p‐CaMKKβ to CaMKKβ (D) (right panel). *P < 0.05. ns, not significant. NC, negative control. Ad, AdipoR1. Figure S5. Exercise improves muscle quality, which is dependent on AdipoR1 and FoxO3a (A) Representative haematoxylin and eosin (HE) staining in transverse gastrocnemius muscles sections from mice of 16‐month‐old and 20‐month‐old. Scale bar, 50 μm. (B) Representative haematoxylin and eosin (HE) staining in transverse gastrocnemius muscle sections of 20‐month‐old mice. Scale bar, 100 μm. (C and D) Muscle fiber cross‐sectional area (CSA) in gastrocnemius muscles of mice (n = 5 per group). *P < 0.05, **P < 0.01, ***P < 0. [file JCSM-14-1789-s005.docx]

**Supplementary figures for “****Physical exercise attenuates age-related muscle atrophy and exhibits anti-aging effects via the adiponectin receptor 1 signaling”**

Yuan-Li Chen, Yi-Cheng Ma, Jie Tang, Dan Zhang, Qiu Zhao, Jian-Jun Liu, Hong-Shu Tang, Jin-Yu Zhang, Guang-Hui He, Chi-Hui Zhong, Yu-Tong Wu, Heng -Ruo Wen，Lan-Qing Ma, & Cheng-Gang Zou

**
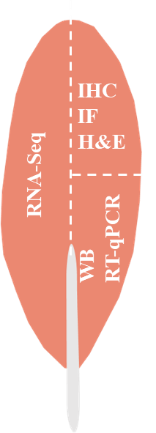
**

**Supplemental Figure 1. The regions of the gastrocnemius muscle used for each assay.**

WB, western blotting; IHC, Immunohistochemical analysis; IF, immunofluorescence staining. H&E, Haematoxylin and eosin (HE) staining.

**
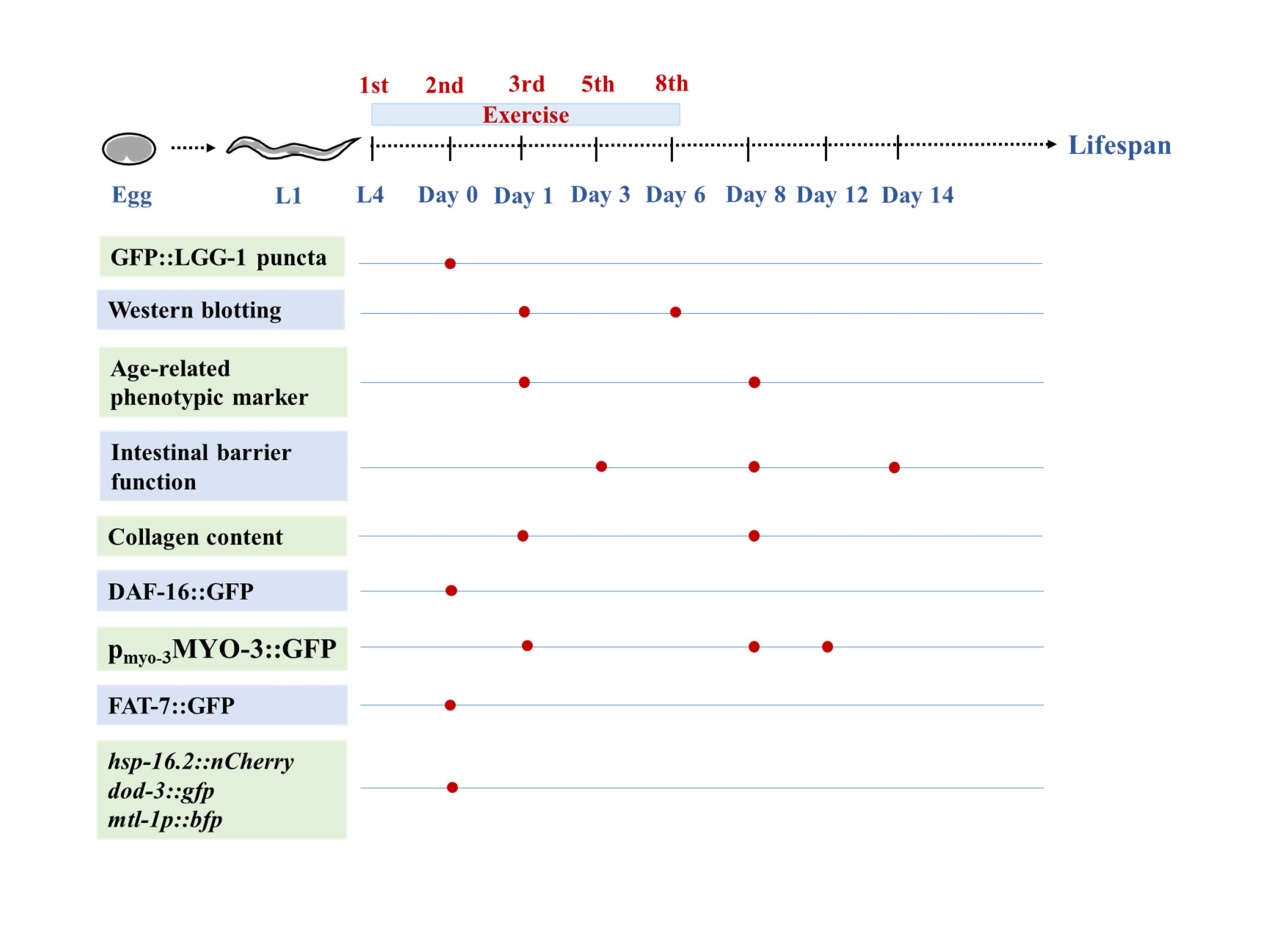
**

**Supplemental Figure 2. Swim exercise regimen for the nematode *C. elegans.***

Starting at the L4 larvae stage, exercise for 90 minutes once a day for eight days. The day when eggs were laid was recorded as day 1 of adulthood.


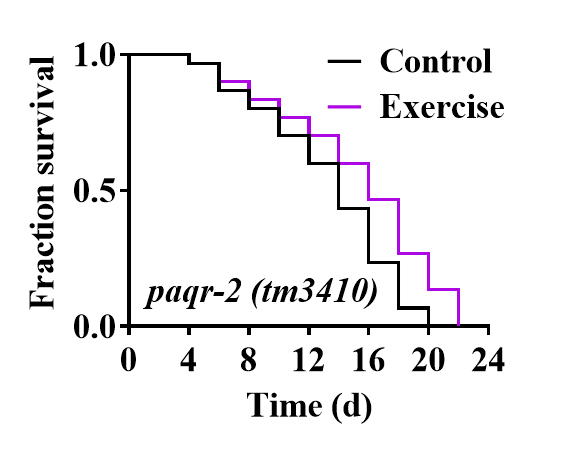


**Supplemental Figure 3. *paqr-2* is not required for exercise-mediated lifespan extension.**

Swim exercise still extended the lifespan in *paqr-2*(*tm3410*) mutants. **P* < 0.05, swim-exercised worms versus control worms.


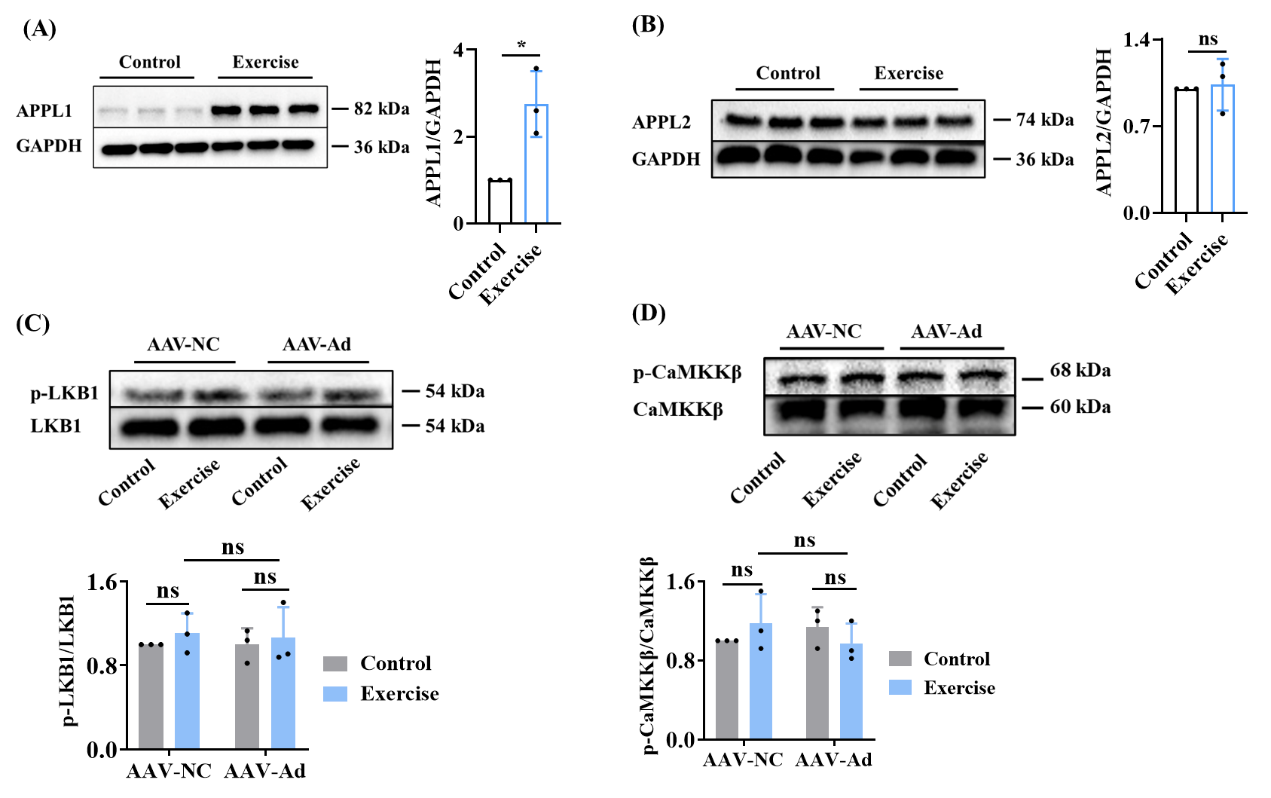


**Supplemental Figure 4. The effect of exercise on the major molecules in the AdipoR1-AMPK signaling.**

(**A and B**) Physical exercise up-regulated the protein levels of APPL1, rather than APPL2, in skeletal muscle of mice. The protein levels of APPL1 (**A**) and APPL2 (**B**) were measured by western blotting (left panel). Quantification of the ratio of APPL1 (**A**) and APPL2 (**B**) to GAPDH (right panel). (**C and D**) Physical exercise did not affect the phosphorylation of LKB1 and CaMKKβ in skeletal muscle of mice. Furthermore, knockdown of AdipoR1 by RNAi did not affect the phosphorylation of LKB1 and CaMKKβ. The levels of phospho-LKB1(Ser428) (**C**) and phospho-CaMKKβ(Ser511) (**D**) were measured by western blotting (left panel). Quantification of the ratio of p-LKB1 to LKB1 (**C**) or p-CaMKKβ to CaMKKβ (**D**) (right panel). **P* < 0.05. ns, not significant. NC, negative control. Ad, AdipoR1.


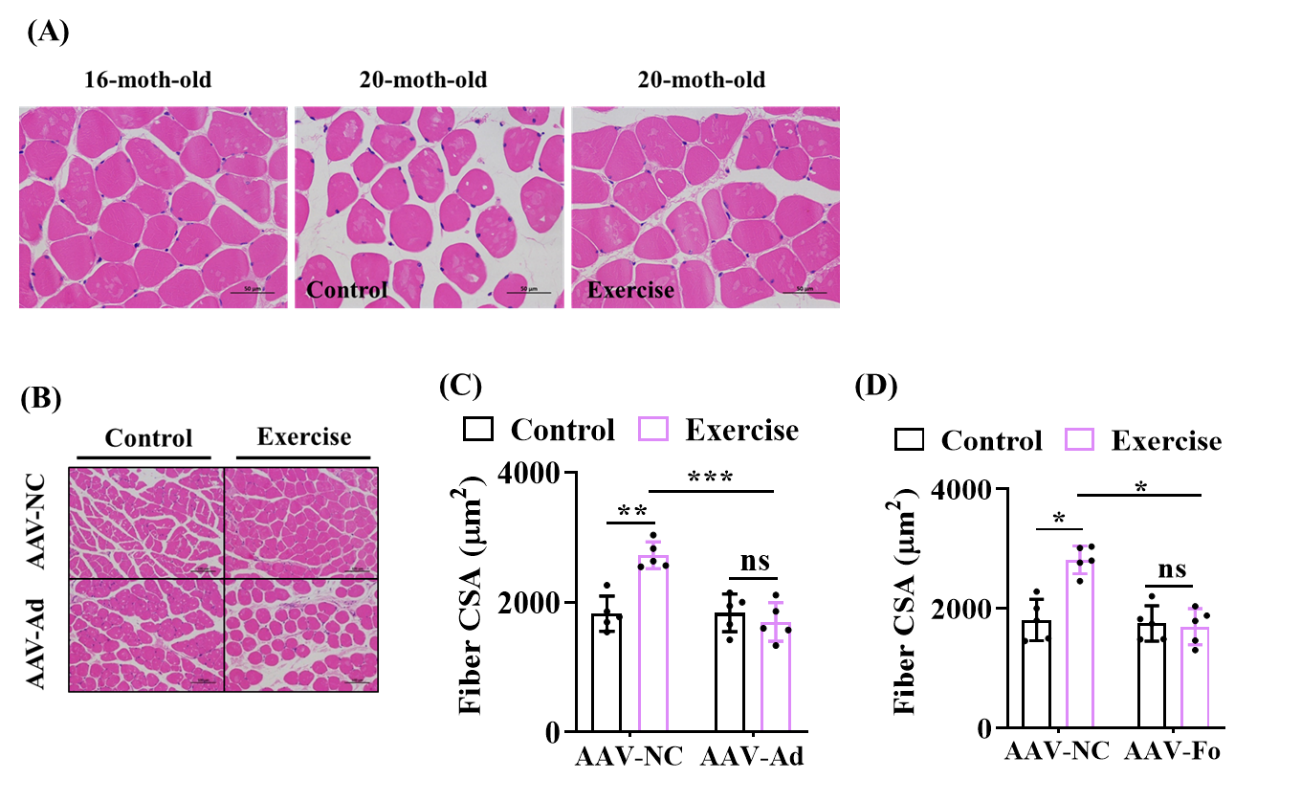


**Supplemental Figure 5. Exercise improves muscle quality, which is dependent on AdipoR1 and FoxO3a**

**(A)** Representative haematoxylin and eosin (HE) staining in transverse gastrocnemius muscles sections from mice of 16-month-old and 20-month-old. Scale bar, 50 μm. (**B**) Representative haematoxylin and eosin (HE) staining in transverse gastrocnemius muscle sections of 20-month-old mice. Scale bar, 100 μm. (**C and D**) Muscle fiber cross-sectional area (CSA) in gastrocnemius muscles of mice (n=5 per group). **P* < 0.05, ***P* < 0.01, ****P* < 0.001, ns, not significant. NC, negative control. Ad, AdipoR1. Fo, FoxO3a.


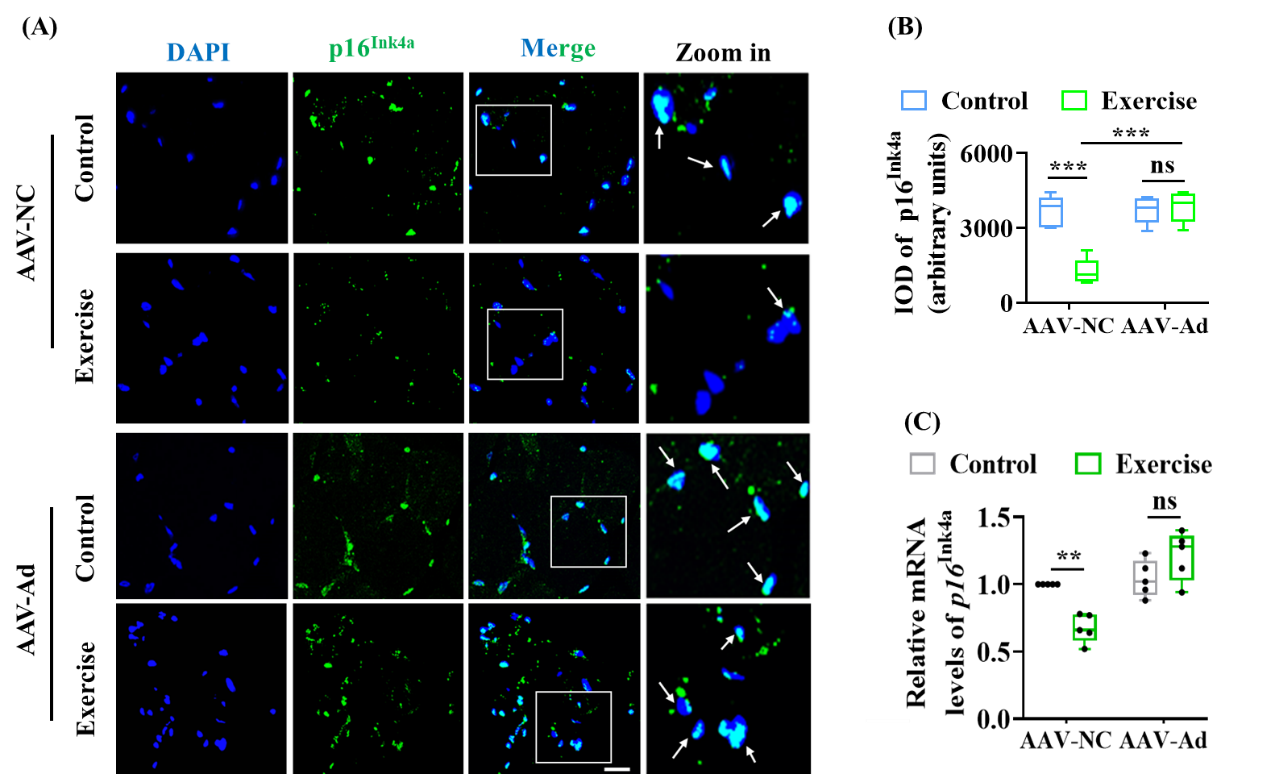


**Supplemental Figure 6. Exercise reduces the expression of p16INK4a via AdipoR1 in skeletal muscle of mice.**

(**A and B**) The protein levels of p16^INK4a^ were reduced in skeletal muscle of exercised mice. This decrease was inhibited by knockdown of AdipoR1 by shRNA. Representative images of immunofluorescence staining for p16^INK4a^ in gastrocnemius muscles of mice. Scale bar, 20 μm **(A)**. Quantification of p16Ink4a protein expression **(B)**. **(C)** Quantitative real-time PCR analysis of *p16^Ink4a^* mRNA expression. ***P* < 0.01, ****P* < 0.01, ns, not significant. NC, negative control. Ad, AdipoR1.


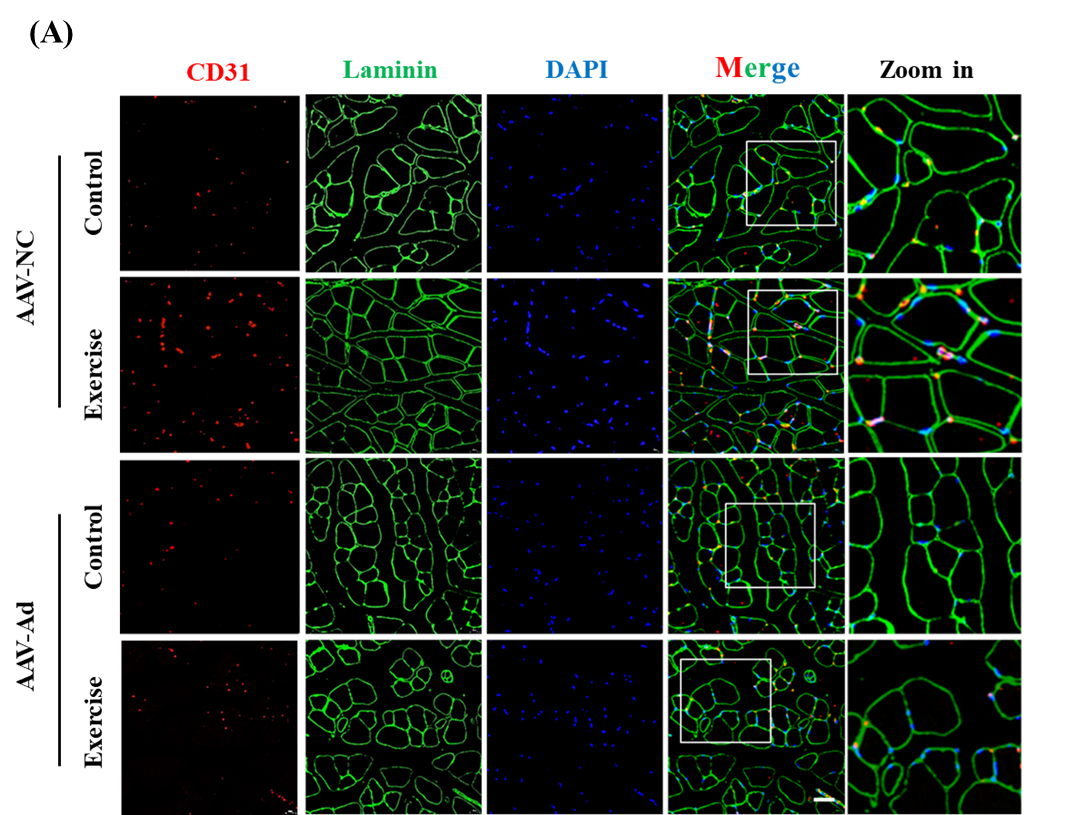


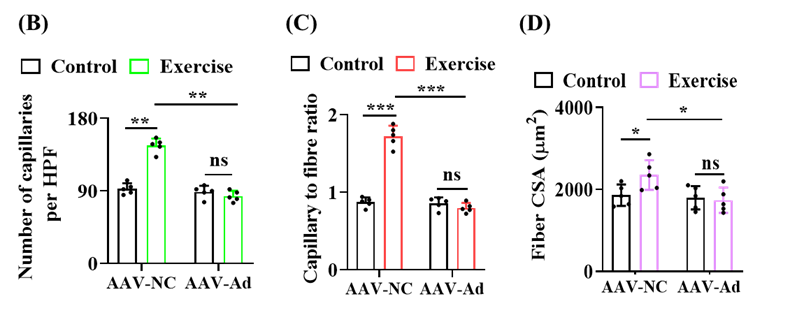


**Supplemental Figure 7. AdipoR1** **is** **involved in the exercise-****induced improvement of muscle** **atrophy.**

**(A)** Representative images of CD31 and Laminin immunofluorescence staining in mice gastrocnemius muscles. NC, negative control. Ad, AdipoR1. Scale bar, 40 μm. **(B-D)** Exercise increased the number of capillaries (**B**), the ratio of capillary to fiber (**C**), and fiber cross-sectional area (CSA) **(D)** in the gastrocnemius muscles. These increases were inhibited by knockdown of AdipoR1 by shRNA. Values are presented as mean ± SD, n=5 per group. **P* < 0.05, ***P* < 0.01, ****P* < 0.01, ns, not significant. NC, negative control. Ad, AdipoR1.


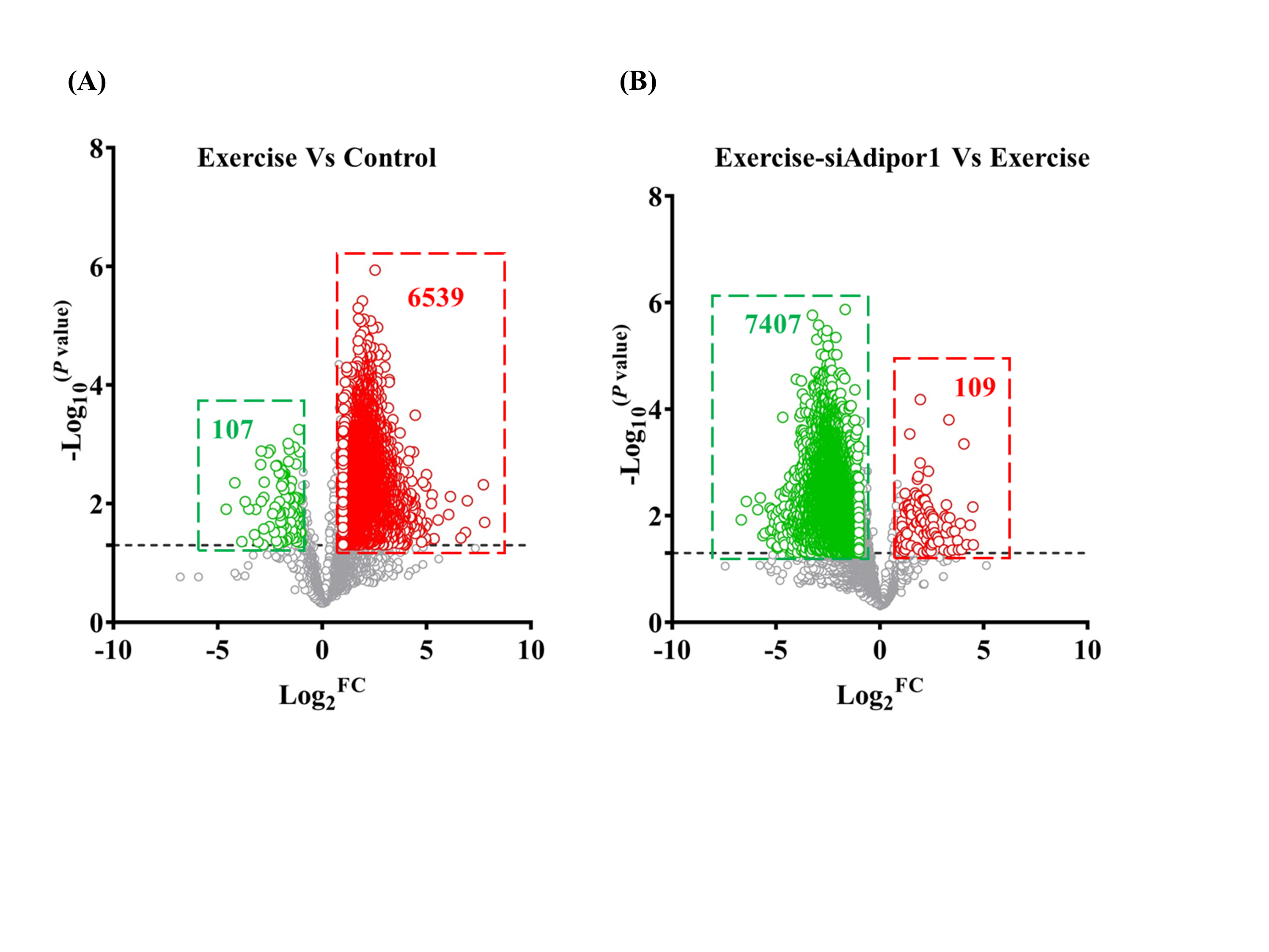
**Supplemental Figure 8. RNA-Seq analysis the transcriptomic profiles of mice after wheel running exercise.**

(**A and B**) Volcano Plot for differential gene expression was performed by comparing exercised mice to control mice (**A**), and exercised mice subjected to AdipoR1 shRNA versus exercised mice (**B**). The red dots represent the significantly upregulated genes and the green dots represent the significantly downregulated genes.


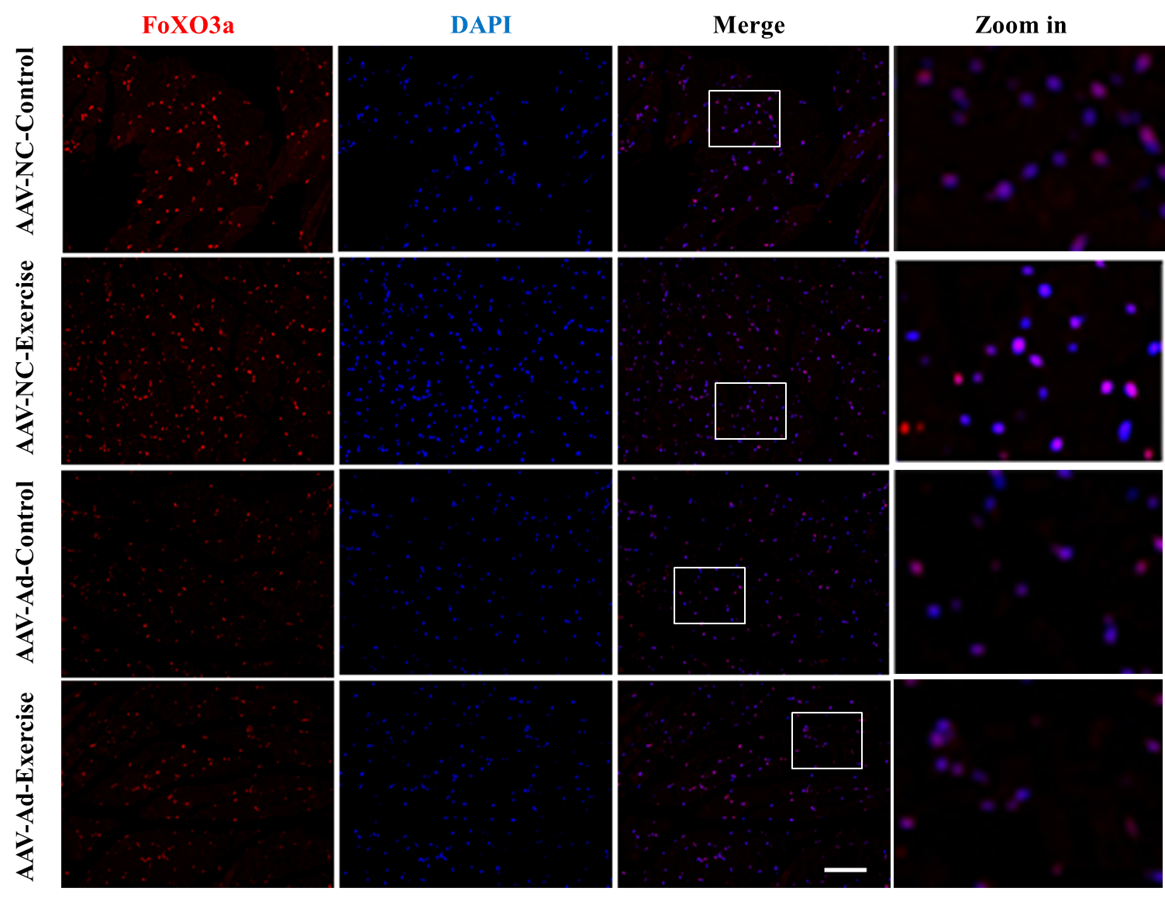


**Supplemental Figure 9. Subcellular localization of FOXO3a in skeletal muscle is not affected by either exercise or AdipoR1 knockdown.**

Representative images of immunofluorescence staining for FOXO3a in gastrocnemius muscles of mice. NC, negative control. Ad, AdipoR1. Scale bar, 100 μm.


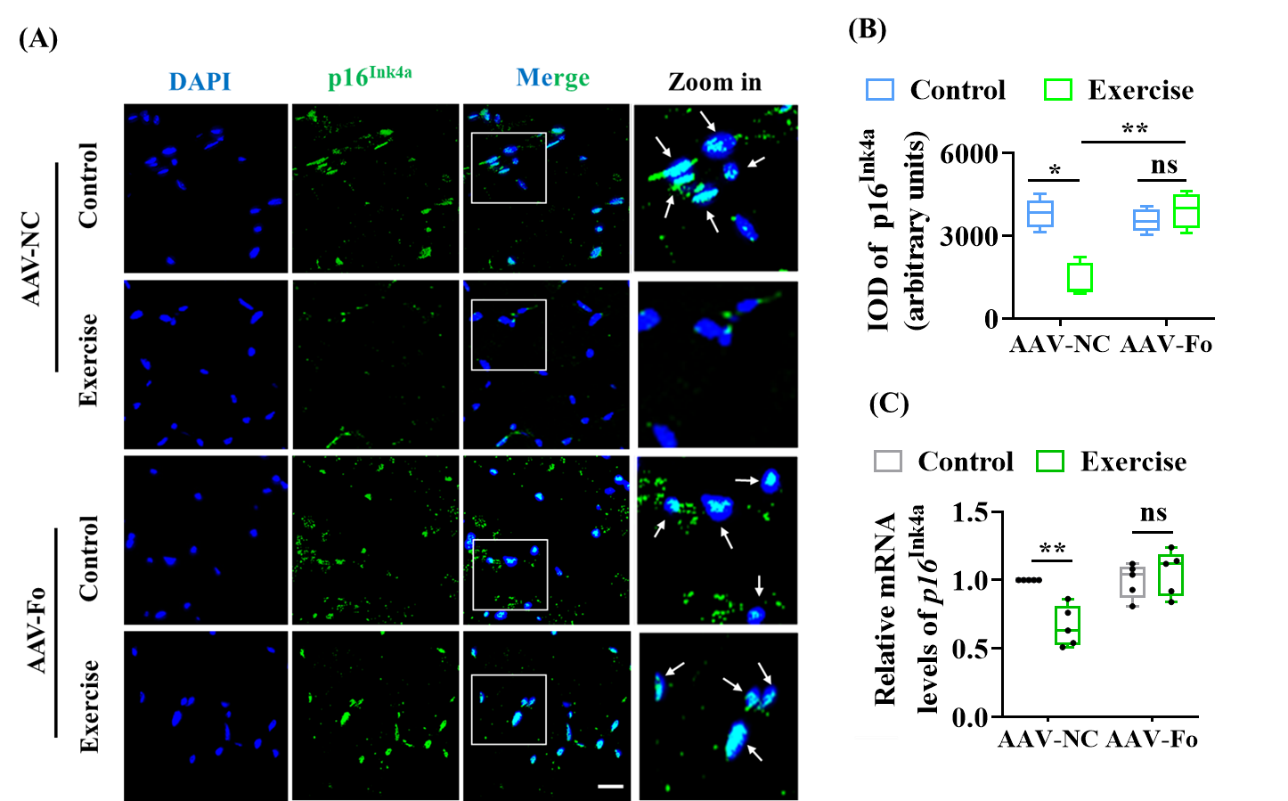


**Supplemental Figure 10. Exercise reduces the expression of p16INK4a via FOXO3a in skeletal muscle of mice.**

(**A and B**) The protein levels of p16^INK4a^ were reduced in skeletal muscle of exercised mice. This decrease was inhibited by knockdown of FoxO3a by shRNA. Representative images of immunofluorescence staining for p16^INK4a^ in gastrocnemius muscles of mice. Scale bar, 20 μm **(A)**. Quantification of p16Ink4a protein expression **(B)**. **(C)** Quantitative real-time PCR analysis of *p16^Ink4a^* mRNA expression. **P* < 0.05, ***P* < 0.01, ns, not significant. NC, negative control. Fo, FoxO3a.

**
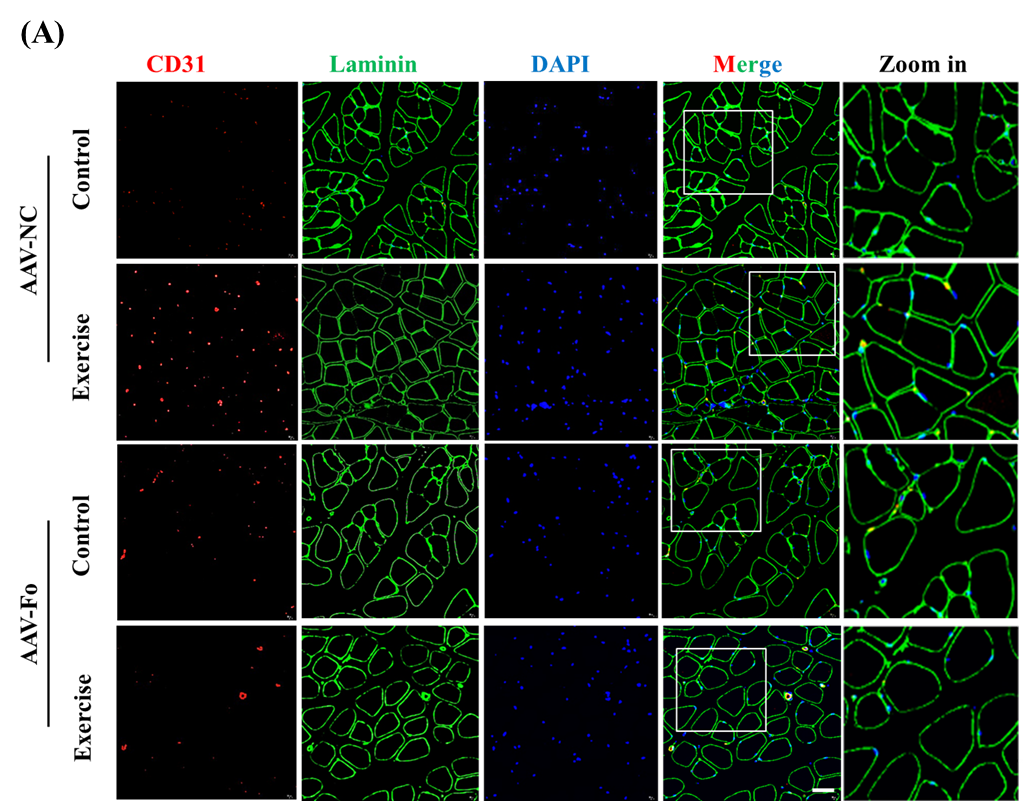
**

**
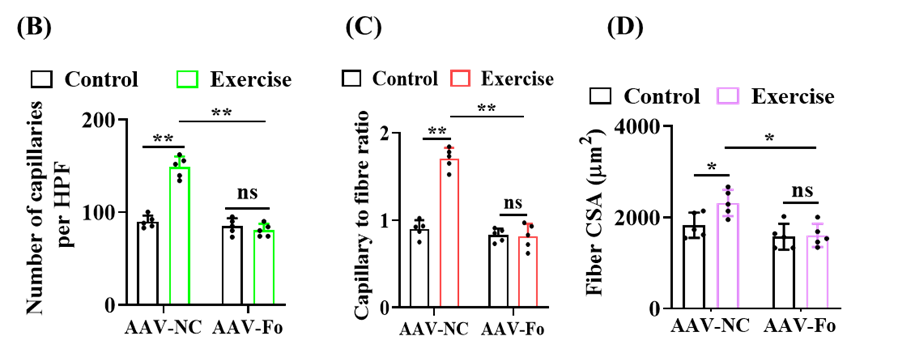
**

**Supplemental Figure 11. Exercise improves number and density of capillaries via FOXO3a in skeletal muscle of mice.**

**(A)** Representative images of immunofluorescence staining for CD31 and Laminin in gastrocnemius muscles of mice, Scale bar, 100 μm. **(B-D)** Exercise increased the number of capillaries (**B**), the ratio of capillary to fiber (**C**), and fiber cross-sectional area (CSA) **(D)** in the gastrocnemius muscles. These increases were inhibited by knockdown of FoxO3a by shRNA. Values are presented as mean ± SD, n=5 per group. **P* < 0.05, ***P* < 0.01, ns, not significant. NC, negative control. Fo, FoxO3a.


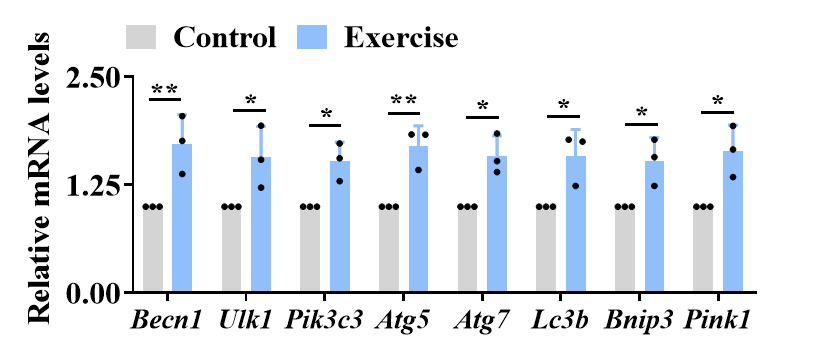


**Supplemental Figure 12. Exercise up-regulates the expression of these autophagy-related genes in worms.**

These results are means ± SD of three independent experiments. **P* < 0.05. ***P* < 0.01,


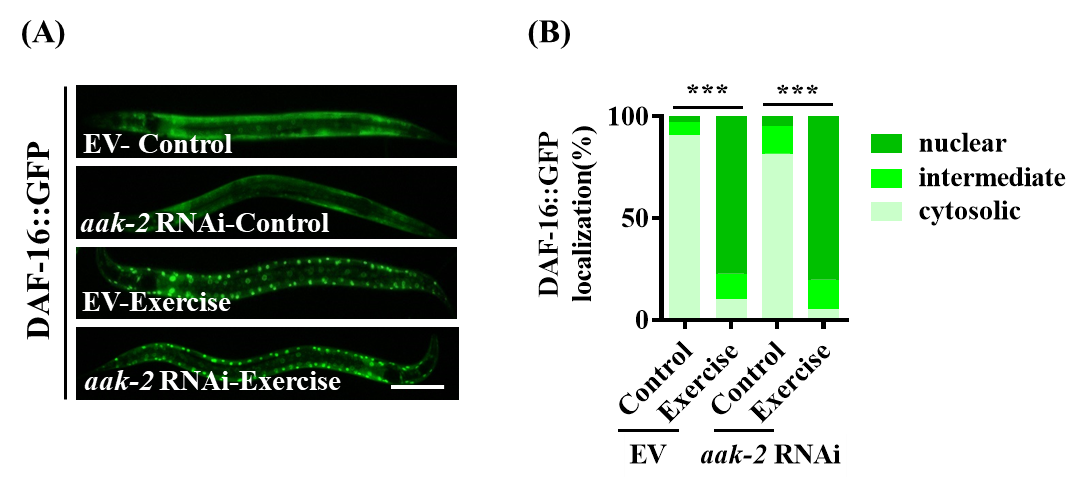


**Supplemental Figure 13. Exercise induces the nuclear accumulation of DAF-16, which is independent of AMPK in worms.**

(**A and B**) Swim exercise significantly increased the nuclear accumulation of DAF-16::GFP. RNAi knockdown of *aak-2* encoding the *C. elegans* AMPK α2 catalytic subunit did not affect the nuclear localization of DAF-16 induced by swim exercise. Representative images of DAF-16::GFP expression pattern in worms (**A**). Scale bars: 100 μm. Quantification of DAF-16 distribution (**B**). ****P* < 0.001.

**
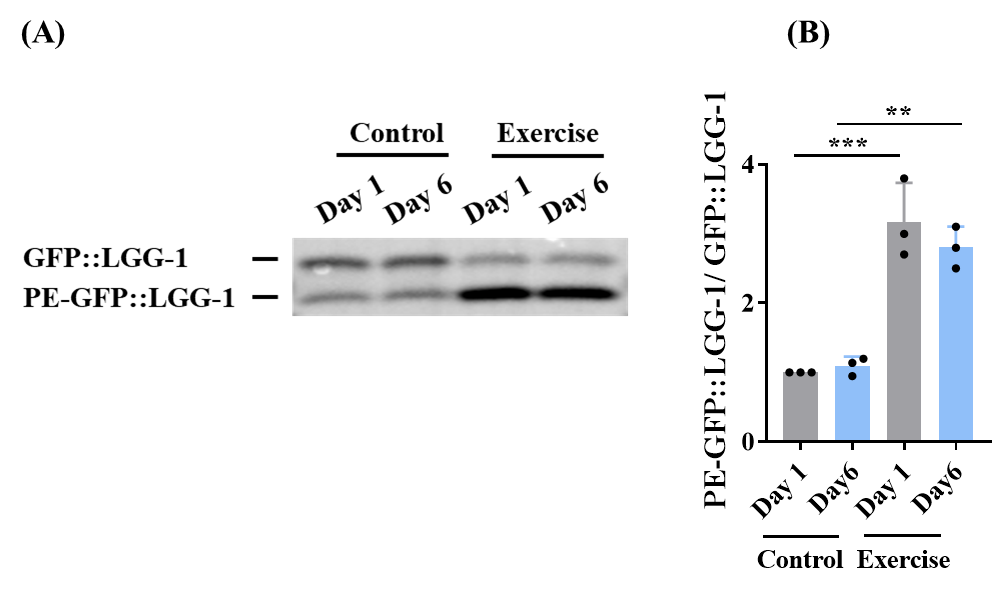
**

**Supplemental Figure 14 Exercise promotes autophagy in worms.**

(**A**) The protein levels of PE-GFP-LGG-1 and GFP-LGG-1 in worms were measured by western blotting. The blot shown here is typical of three independent experiments. (**B**) Quantification of the ratio of PE-GFP-LGG-1 to GFP-LGG-1. These results are means ± SD of three independent experiments. ***P* < 0.01, ****P* < 0.001.

**.
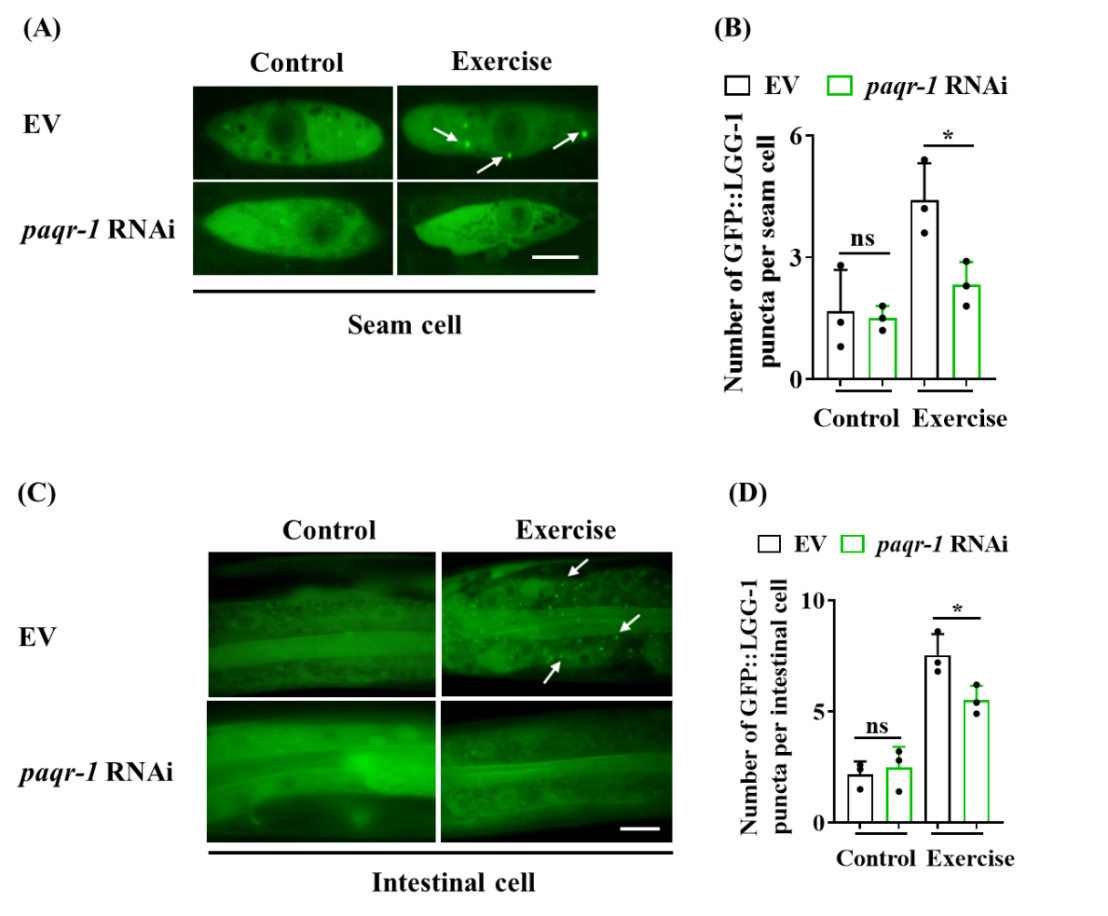
 Supplemental Figure 15. PAQR-1 is** **involved in autophagy induction in exercised worms.**

**(A)** Representative images of autophagosomes (GFP::LGG-1 puncta) in the seam cells of control worms and swim-exercised worms, respectively. The arrow denotes a representative autophagosome. Scale bars: 10 μm. **(B)** The numbers of GFP::LGG-1 puncta were counted in the seam cells. Values are presented as mean ± SD of three independent experiments (n = 30-35 worms per experiment). **(C)** Representative images of autophagosomes (GFP::LGG-1 puncta) in the intestinal cells of control worms and swim-exercised worms, respectively. The arrow denotes a representative autophagosome. Scale bars: 10 μm. **(D)** The numbers of GFP::LGG-1 puncta were counted in the intestinal cells. Values are presented as mean ± SD of three independent experiments (n = 30-35 worms per experiment). **P* < 0.05, exercise group versus control group. ns, not significant.


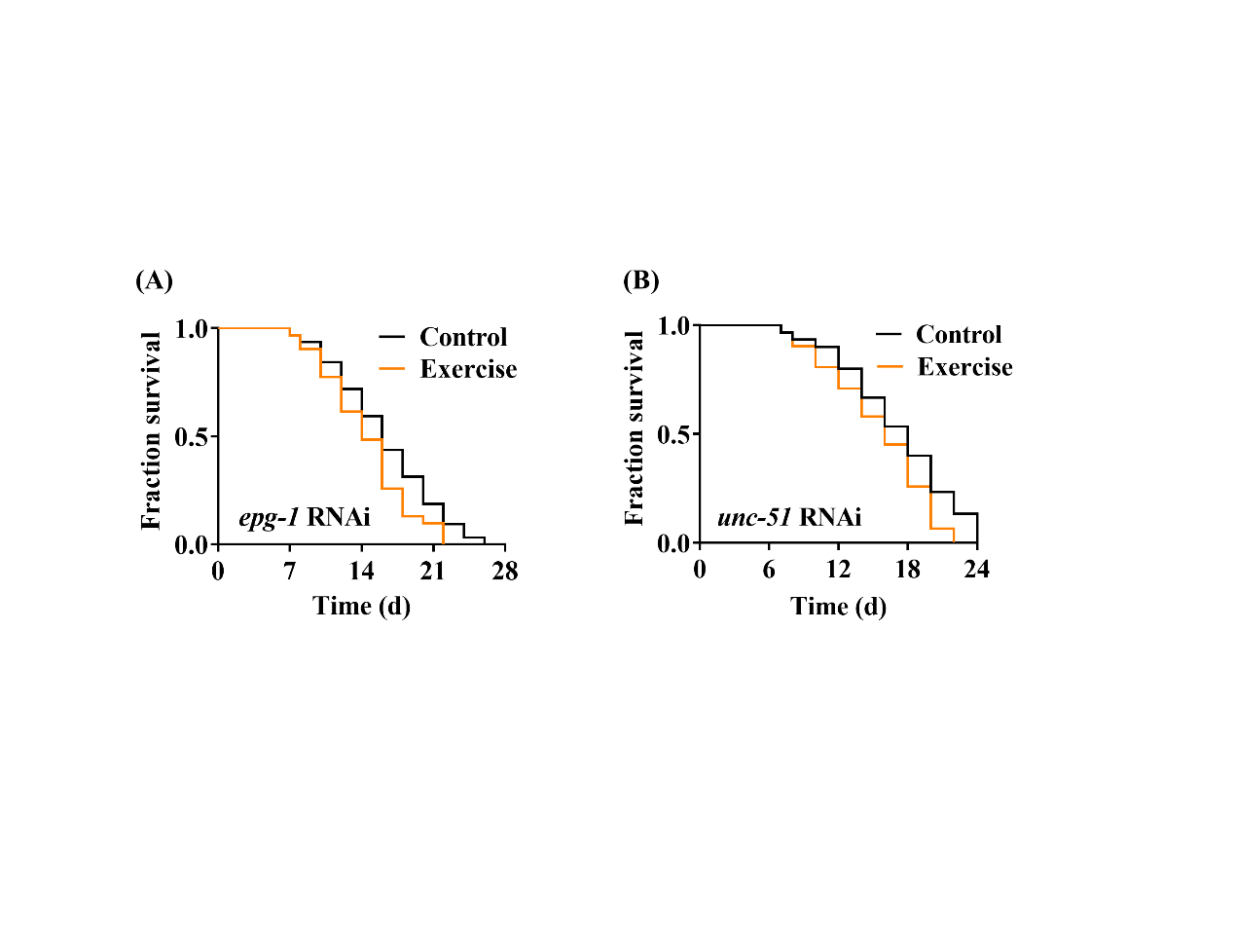


**Supplemental Figure 16. Autophagy-related genes are involved in exercise-induced longevity in worms.**

**(A and B)** Swim exercise failed to extend lifespan in worms subjected to *epg-1***(A)** or *unc-51* **(B)** RNAi, respectively. ns, not significant, exercise group versus control group


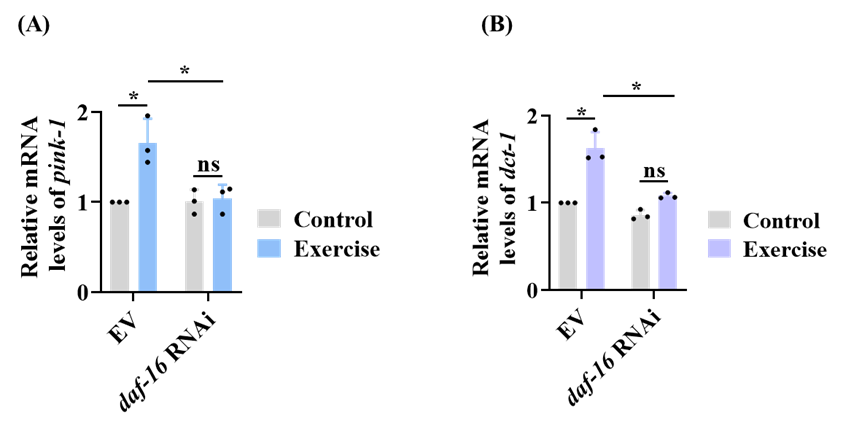


**Supplemental Figure 17. The mRNA levels of *pink-1* and *dct-1* are decreased in swim exercised-worms subjected to daf-16 RNAi.**

**(A and B)** Quantitative real-time PCR analysis of *pink-1***(A)** or *dct-1***(B)** mRNA expression of control worms and swim-exercised worms, respectively. Values are presented as mean ± SD, n=5 per group, **P* < 0.05. ns, not significant.


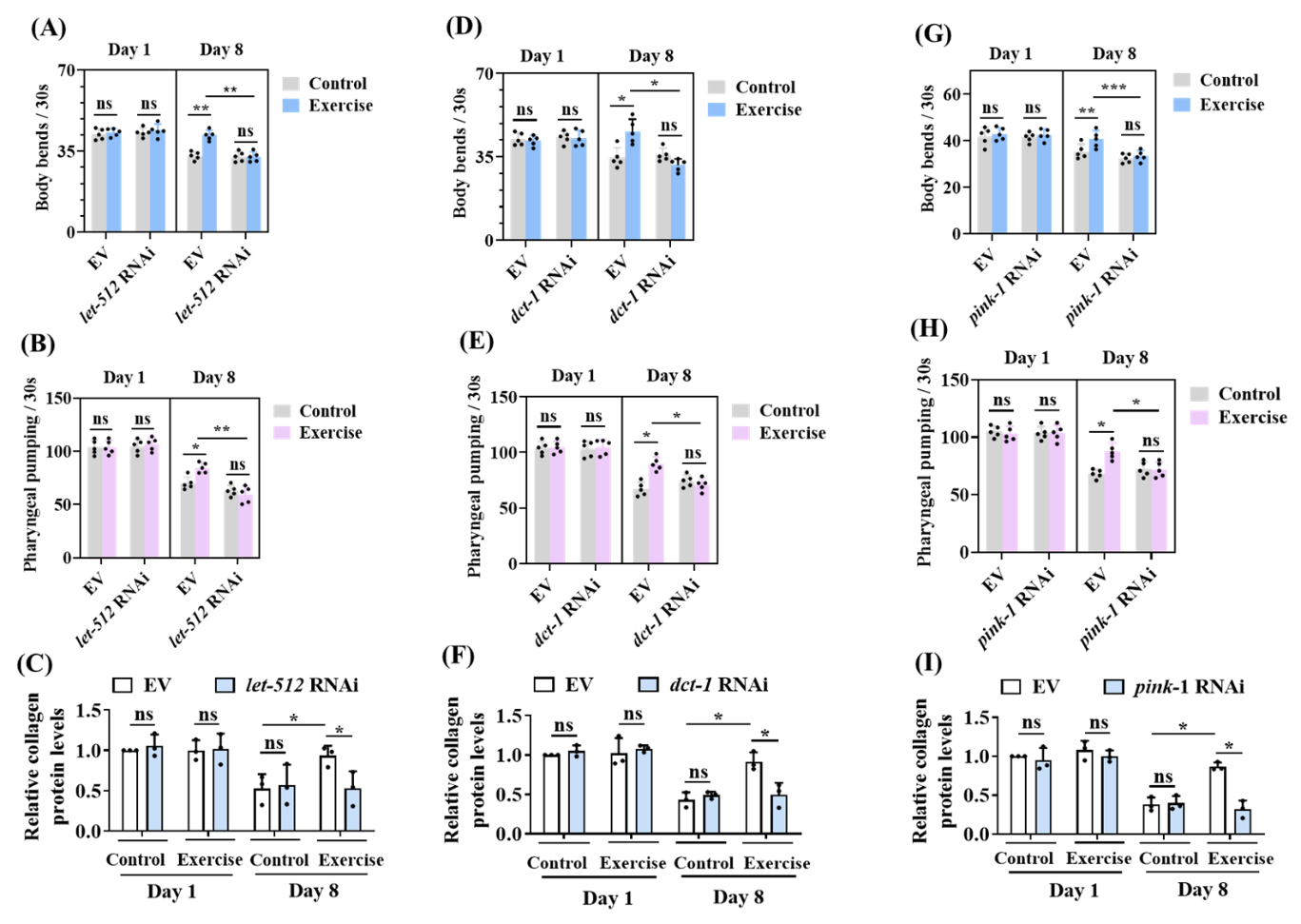


**Supplemental Figure 18. Knockdown of *let-512*, *dct-1* and *pink-1* by RNAi abolishes the beneficial effect of exercise on aging biomarkers in worms.**

**(A-C)** *let-512* was involved in delaying the appearance of the aging markers, including body bending **(A)** pharyngeal pumping **(B)** and relative collagen levels **(C)**, in swim-exercised worms. Values are presented as mean ± SD of three or five independent experiments (n = 30-35 worms per experiment). **(D-F)** *dct-1* was involved in delaying the appearance of the aging markers, including body bending **(D)** pharyngeal pumping **(E)** and relative collagen levels **(F)**, in swim-exercised worms. Values are presented as mean ± SD of three or five independent experiments (n = 30-35 worms per experiment). **(G-I)** *pink-1* was involved in delaying the appearance of the aging markers, including body bending **(G)** pharyngeal pumping **(H)** and relative collagen levels **(I)**, in swim-exercised worms. Values are presented as mean ± SD of three or five independent experiments (n = 30-35 worms per experiment). **P* < 0.05, ***P* < 0.01, ****P* < 0.001, ns, not significant.
