# Supplementary material for: Physical exercise attenuates age‐related muscle atrophy and exhibits anti‐ageing effects via the adiponectin receptor 1 signalling
Source: J Cachexia Sarcopenia Muscle. 2023 May 24;14(4):1789–801. doi: 10.1002/jcsm.13257 (PMC10401527; doi:10.1002/jcsm.13257)
Supplement: Supplementary file 2 — Data S1. Supporting Information [file JCSM-14-1789-s008.pptx]

## Slide 1
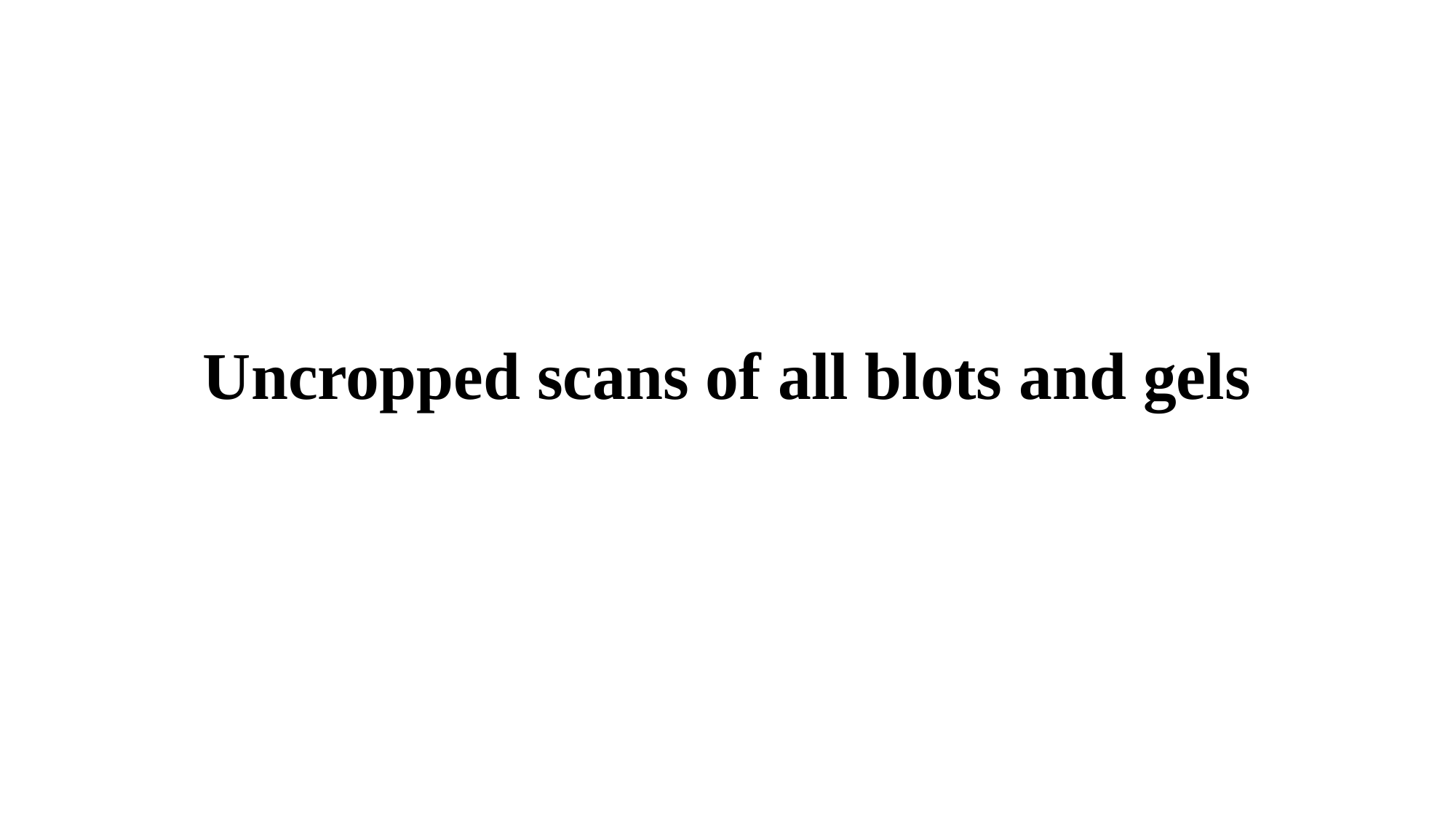

# Uncropped scans of all blots and gels

## Slide 2
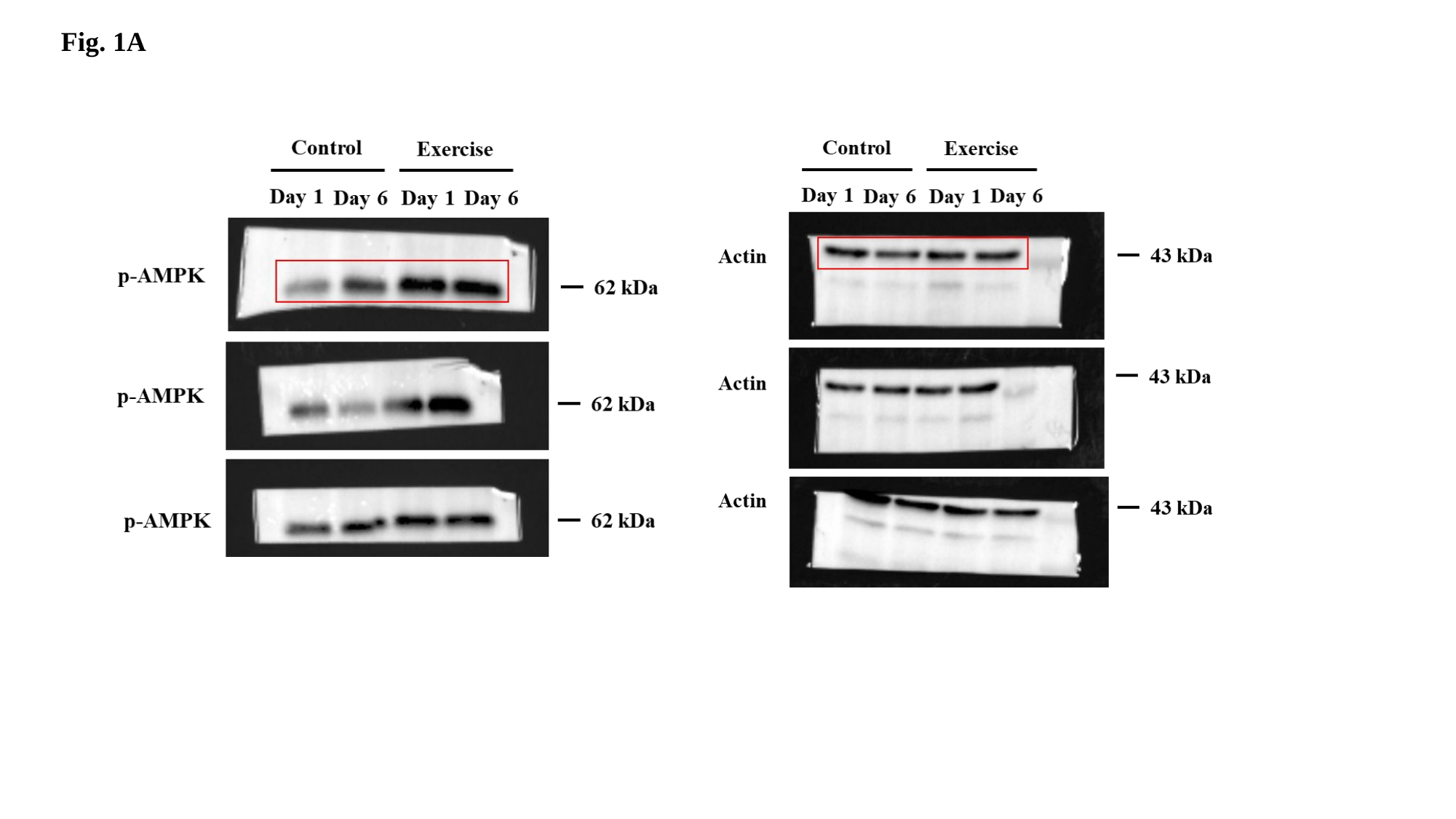

Fig. 1A

## Slide 3
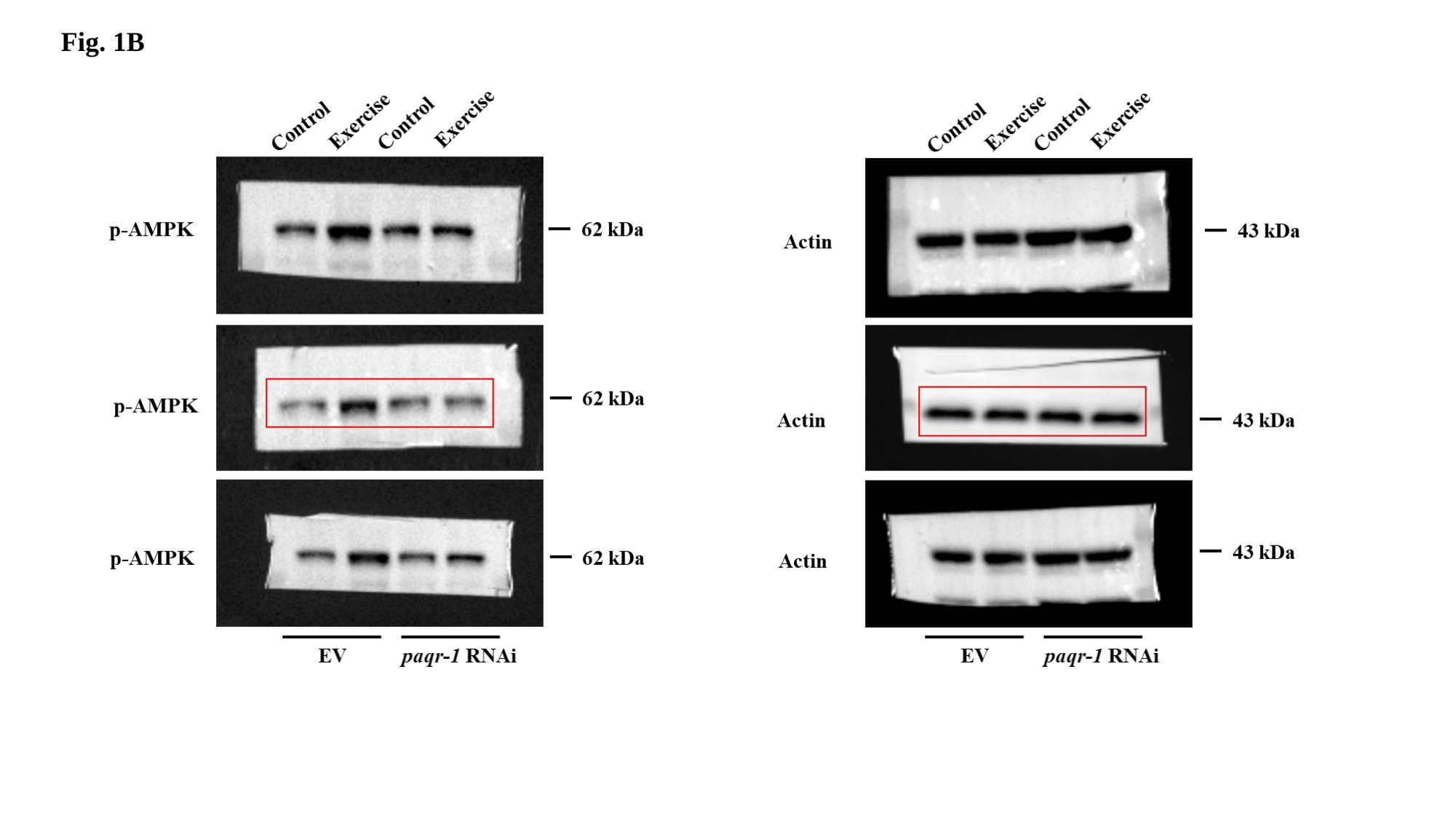

Fig. 1B

## Slide 4
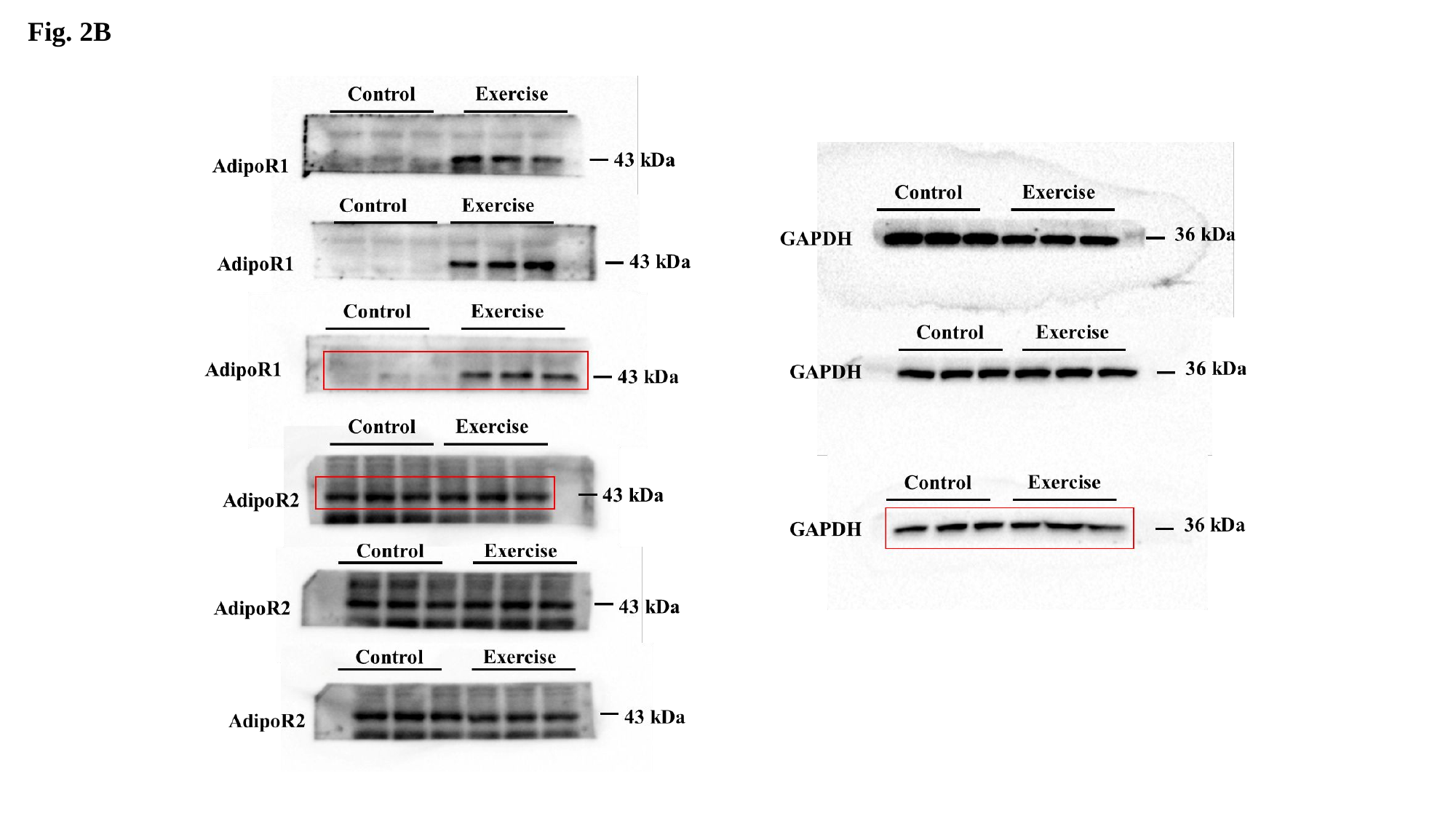

Fig. 2B

## Slide 5
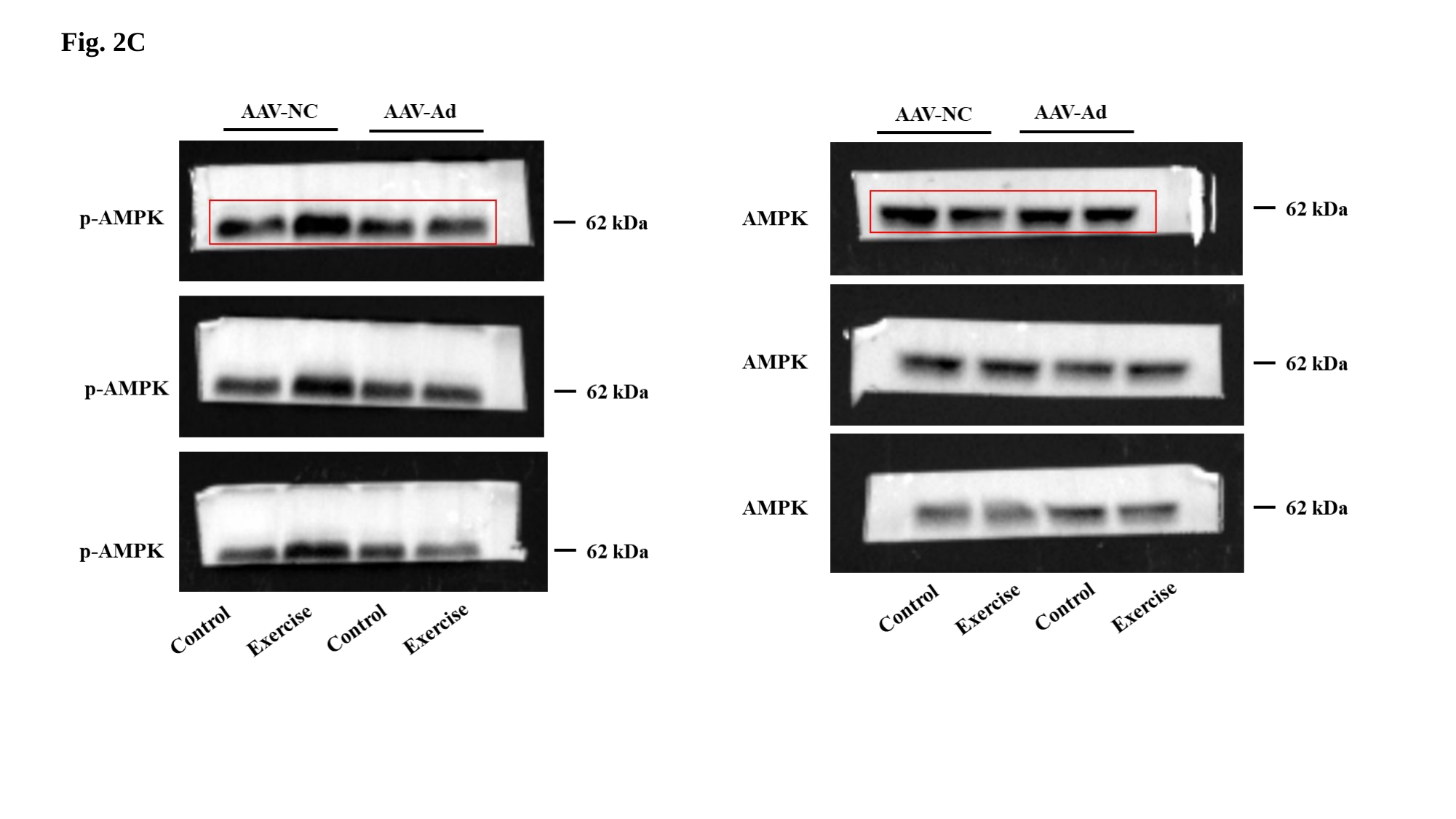

Fig. 2C

## Slide 6
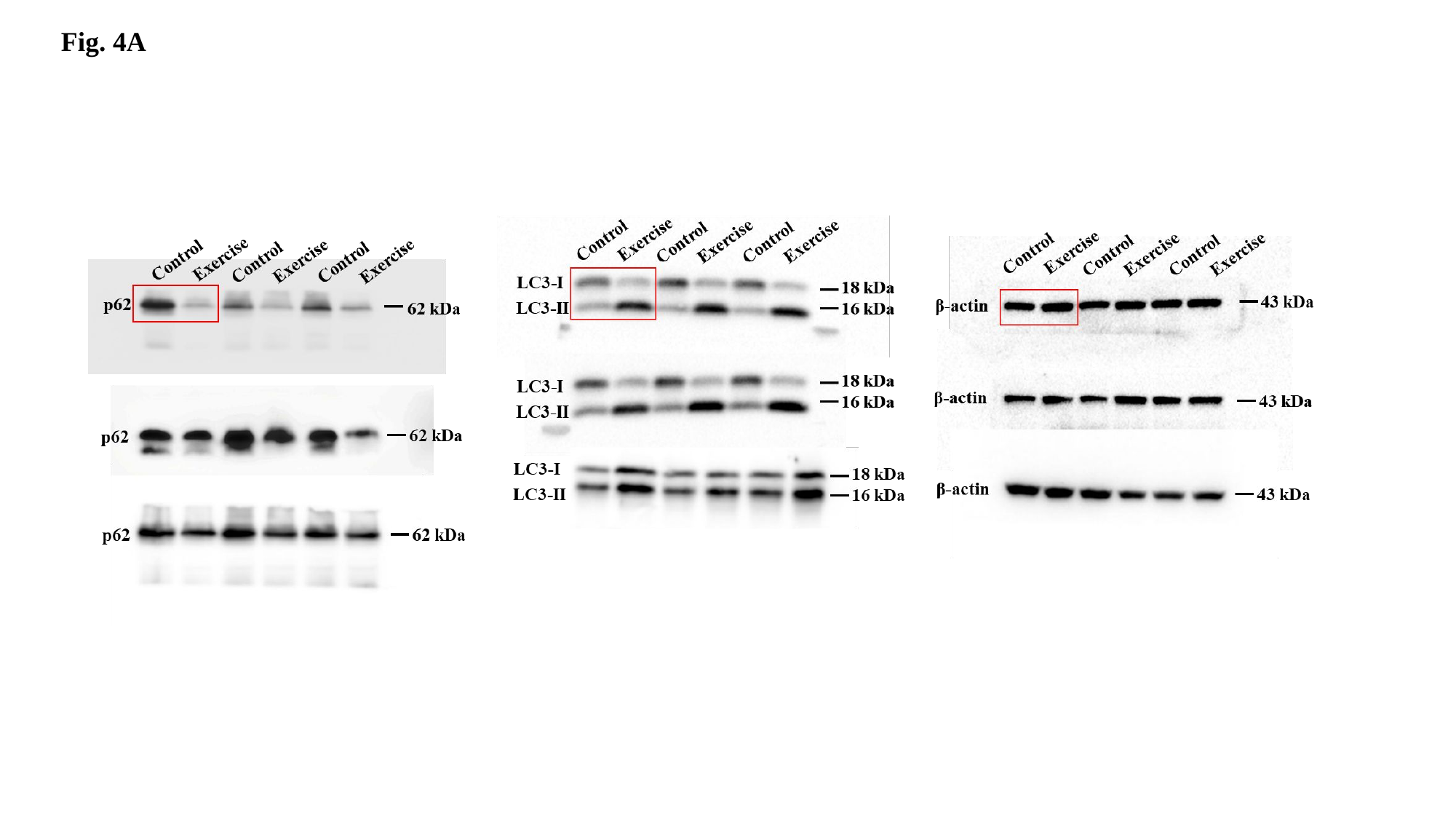

Fig. 4A

## Slide 7
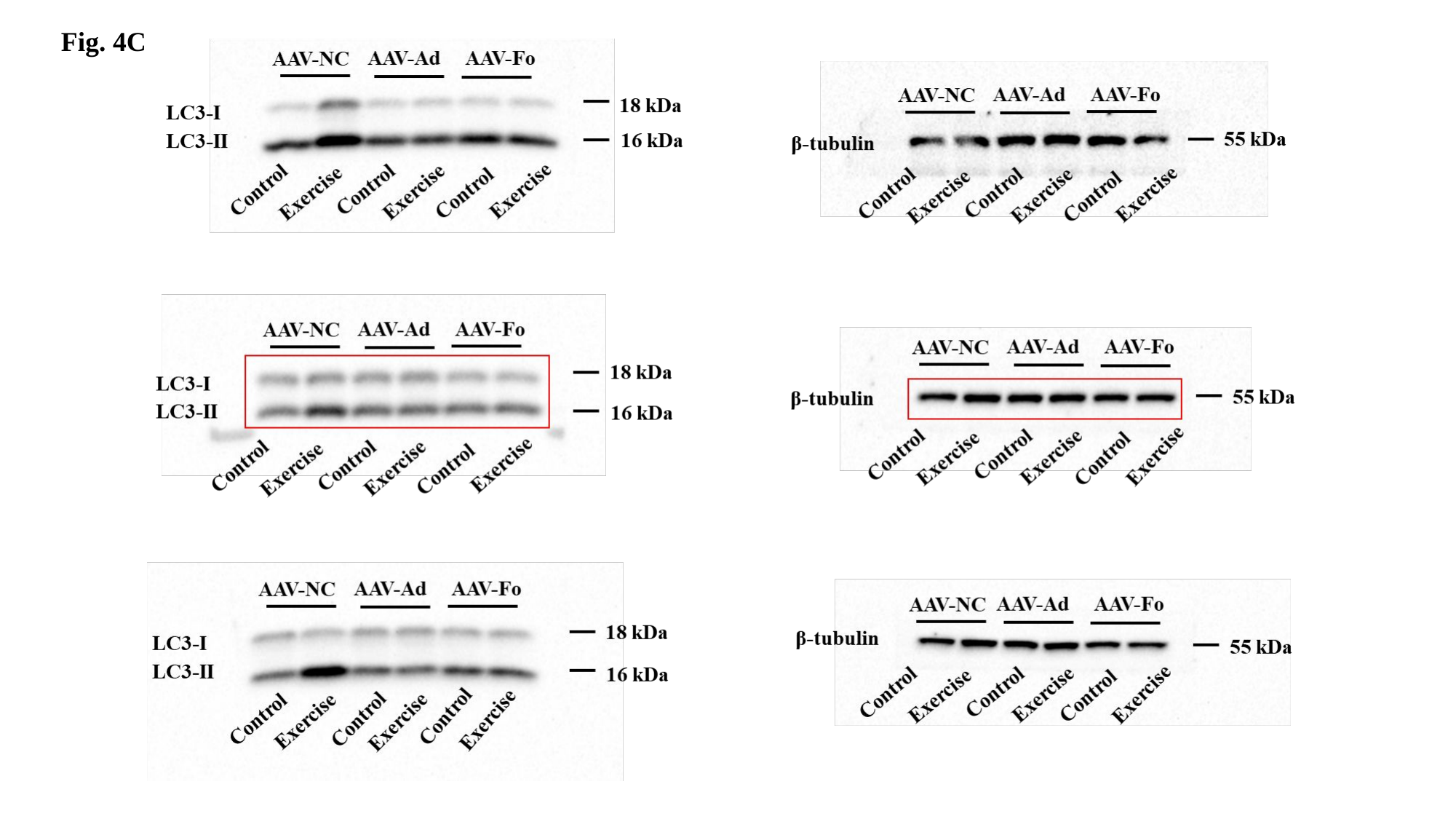

Fig. 4C

## Slide 8
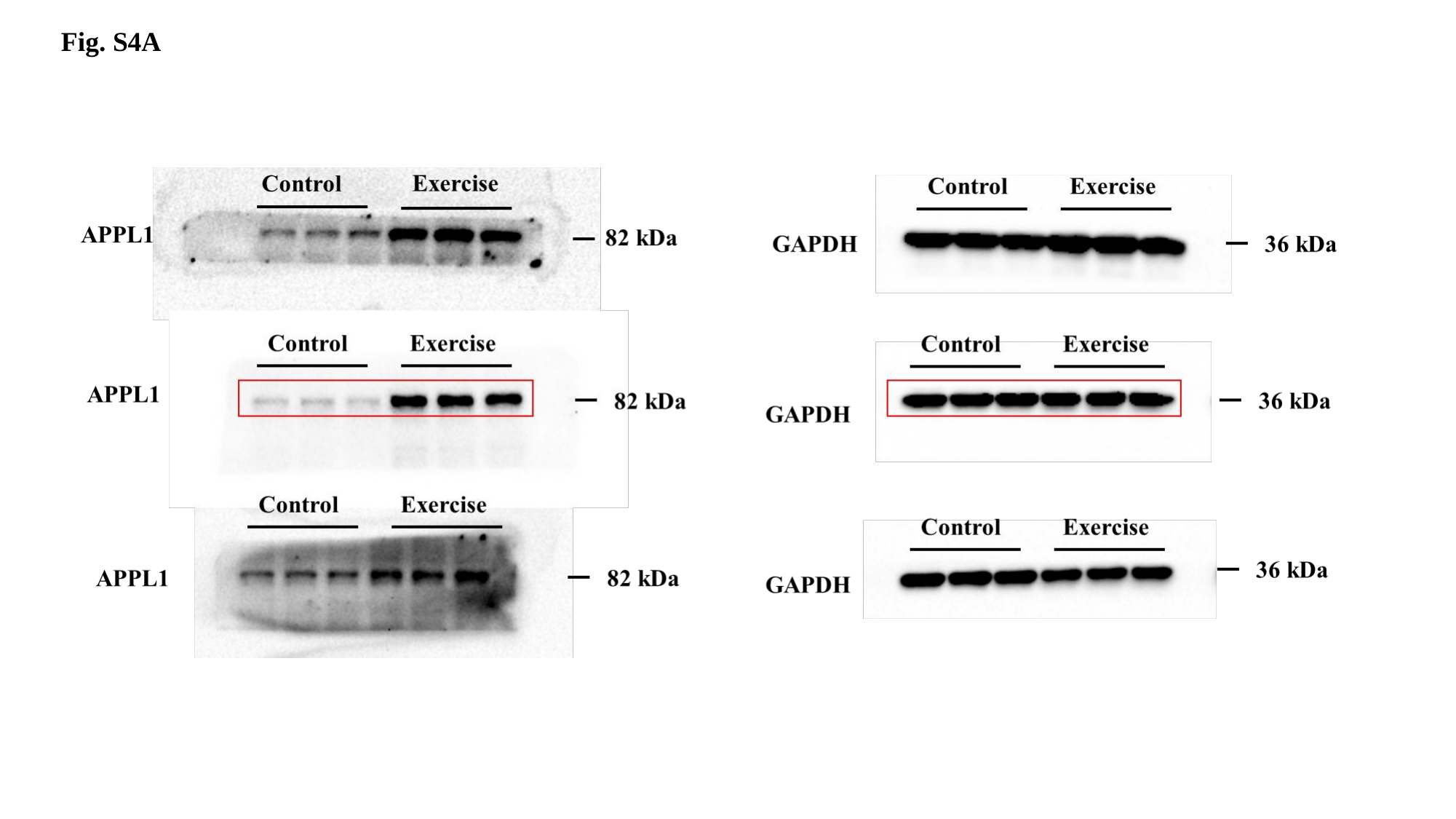

Fig. S4A

## Slide 9
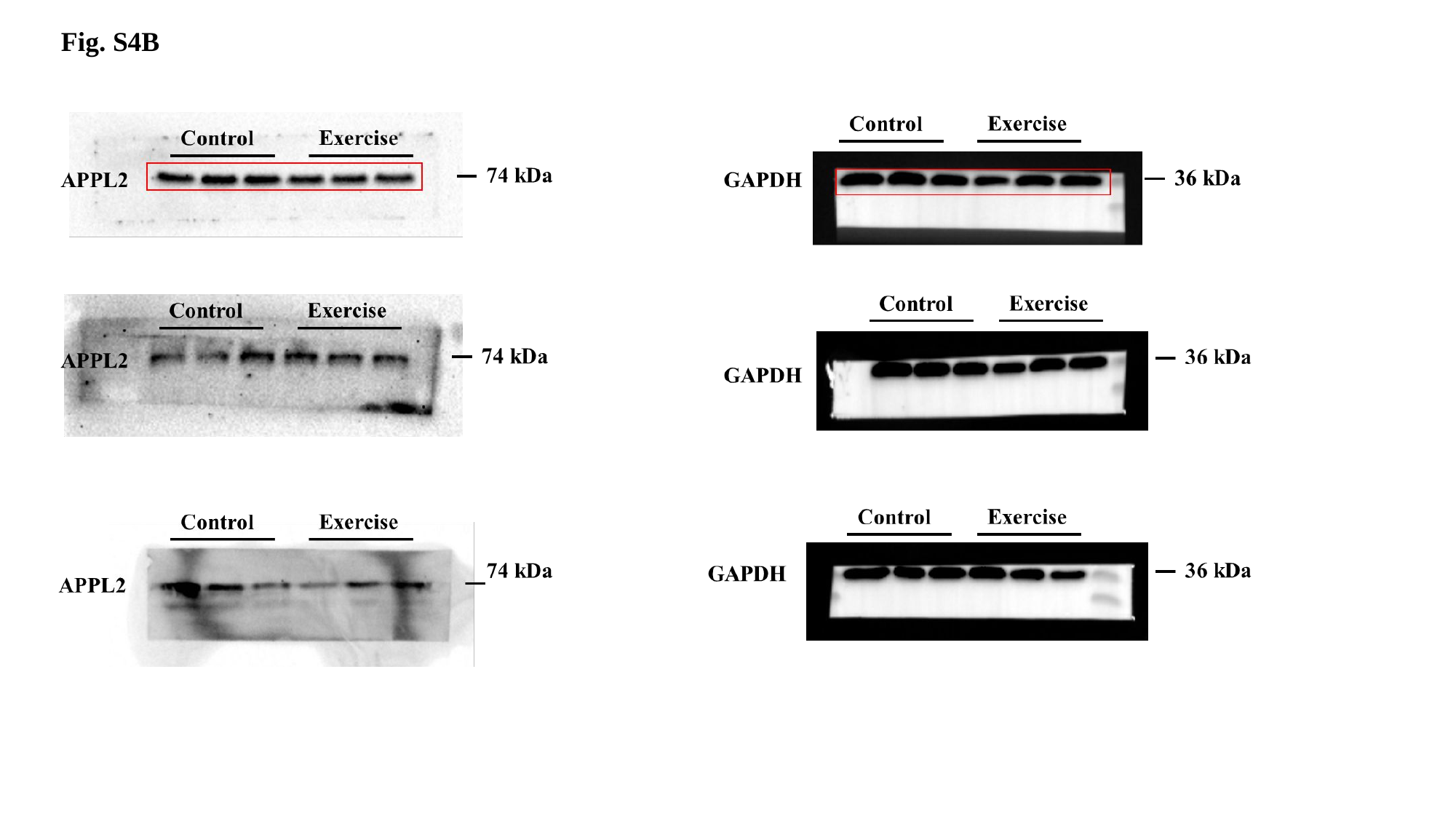

Fig. S4B

## Slide 10
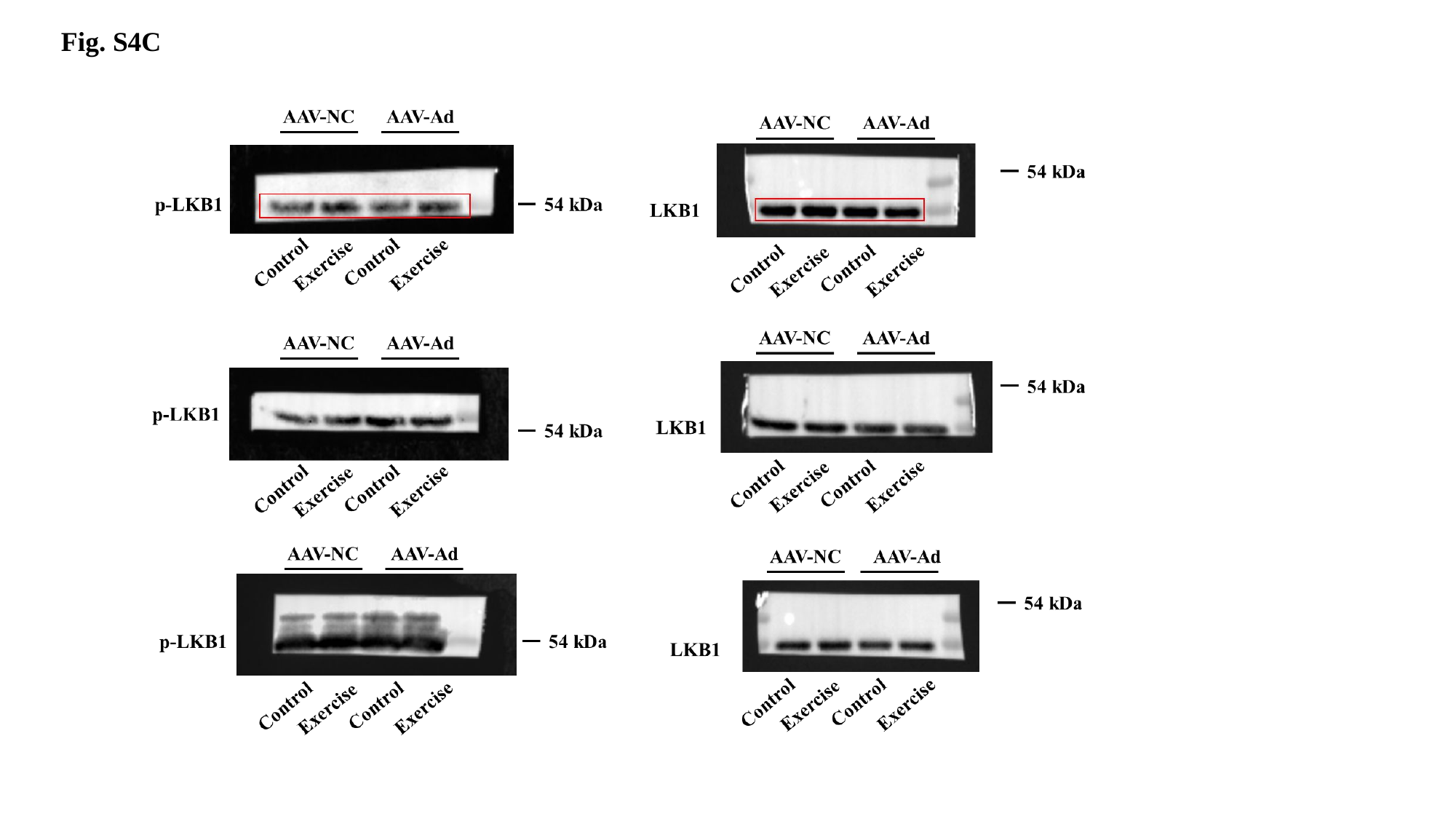

Fig. S4C

## Slide 11
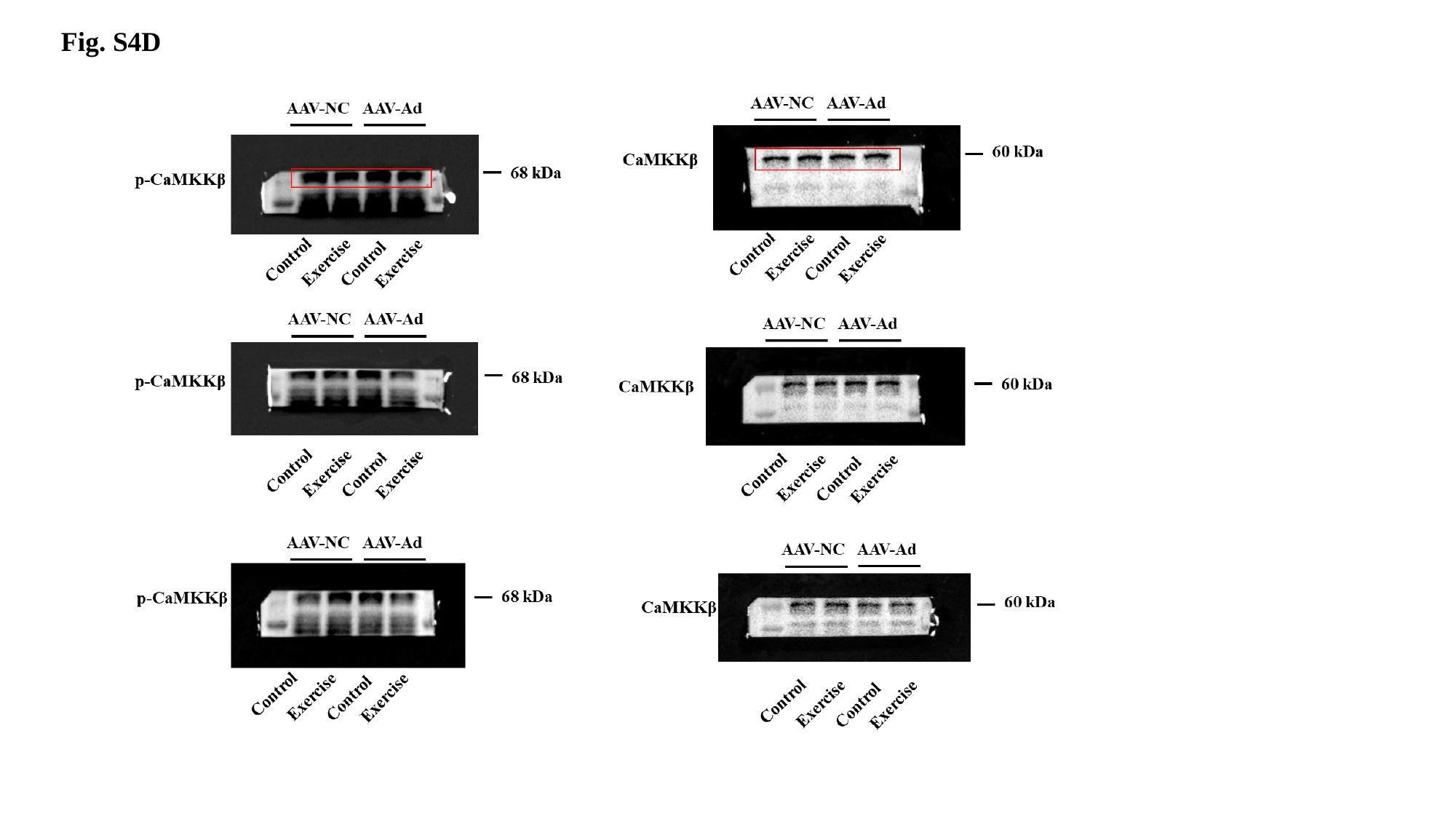

Fig. S4D

## Slide 12
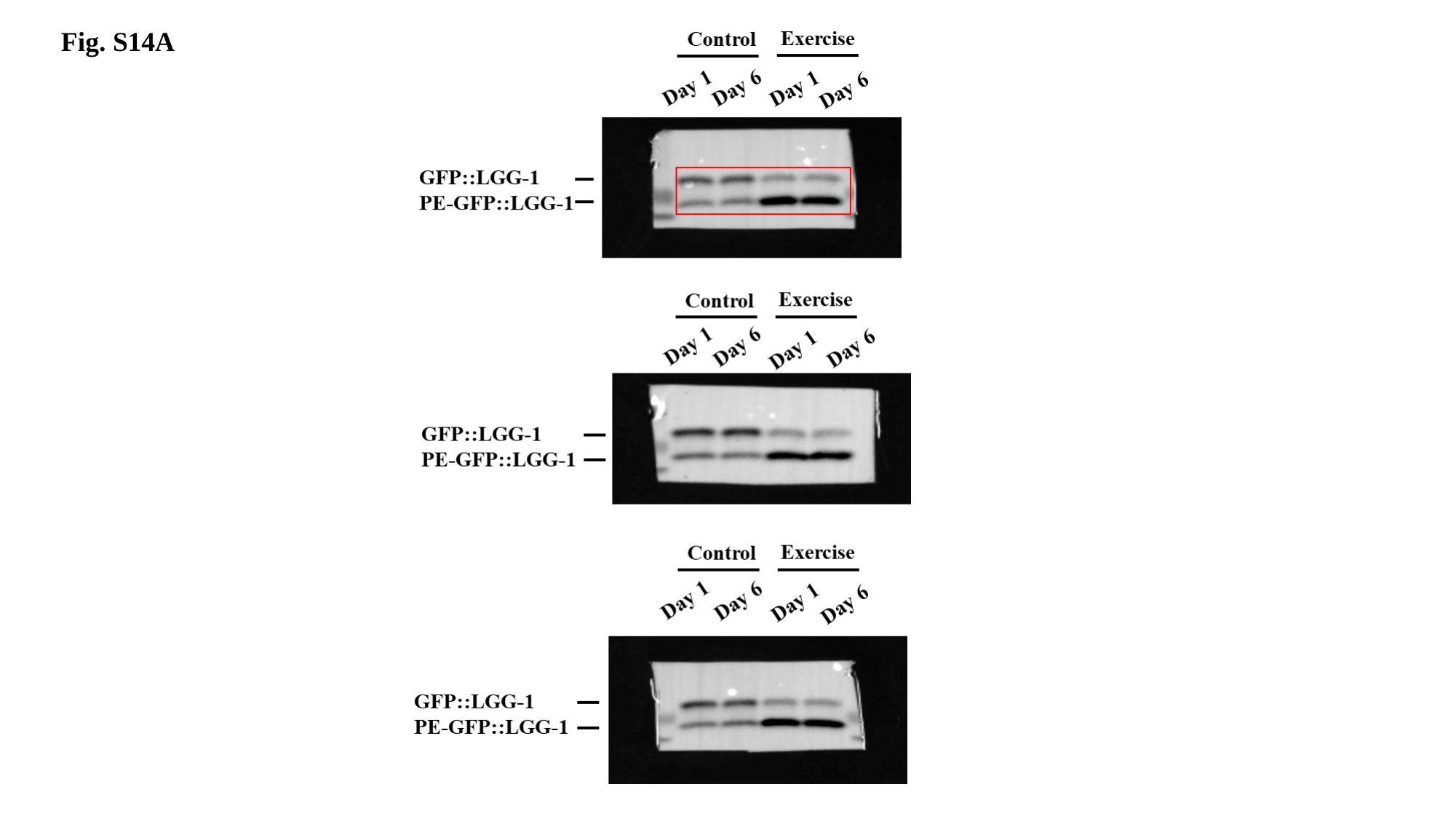

Fig. S14A
